# A Single Residue within the MCR-1 Protein Confers Anticipatory Resilience

**DOI:** 10.1128/spectrum.03592-22

**Published:** 2023-04-18

**Authors:** Renate Frantz, Konrad Gwozdzinski, Nicolas Gisch, Swapnil Prakash Doijad, Martina Hudel, Maria Wille, Mobarak Abu Mraheil, Dominik Schwudke, Can Imirzalioglu, Linda Falgenhauer, Michael Ehrmann, Trinad Chakraborty

**Affiliations:** a Institute of Medical Microbiology, Justus Liebig University Giessen, Giessen, Germany; b Institute of Hygiene and Environmental Medicine, Justus Liebig University Giessen, Giessen, Germany; c Hessian University Competence Center for Hospital Hygiene, Justus Liebig University Giessen, Giessen, Germany; d German Center for Infection Research, Partner Site: Giessen-Marburg-Langen, Giessen, Germany; e Division of Bioanalytical Chemistry, Priority Area Infections, Research Center Borstel, Leibniz Lung Center, Borstel, Germany; f German Center for Infection Research, Partner Site: Hamburg-Lübeck-Borstel-Riems, Borstel, Germany; g Airway Research Center North, Partner Site: Research Center Borstel, Borstel, Germany; h Center of Medical Biotechnology, Faculty of Biology, University Duisburg-Essen, Essen, Germany; i School of Biosciences, Cardiff University, Cardiff, United Kingdom; The Pennsylvania State University

**Keywords:** *E. coli*, MCR-1, envelope stress response (ESR), pH-enhanced resistance, plasmid elimination, lipid A, mass spectrometry

## Abstract

The envelope stress response (ESR) of Gram-negative enteric bacteria senses fluctuations in nutrient availability and environmental changes to avert damage and promote survival. It has a protective role toward antimicrobials, but direct interactions between ESR components and antibiotic resistance genes have not been demonstrated. Here, we report interactions between a central regulator of ESR *viz.*, the two-component signal transduction system CpxRA (conjugative pilus expression), and the recently described mobile colistin resistance protein (MCR-1). Purified MCR-1 is specifically cleaved within its highly conserved periplasmic bridge element, which links its N-terminal transmembrane domain with the C-terminal active-site periplasmic domain, by the CpxRA-regulated serine endoprotease DegP. Recombinant strains harboring cleavage site mutations in MCR-1 are either protease resistant or degradation susceptible, with widely differing consequences for colistin resistance. Transfer of the gene encoding a degradation-susceptible mutant to strains that lack either DegP or its regulator CpxRA restores expression and colistin resistance. MCR-1 production in Escherichia coli imposes growth restriction in strains lacking either DegP or CpxRA, effects that are reversed by transactive expression of DegP. Excipient allosteric activation of the DegP protease specifically inhibits growth of isolates carrying *mcr-1* plasmids. As CpxRA directly senses acidification, growth of strains at moderately low pH dramatically increases both MCR-1-dependent phosphoethanolamine (PEA) modification of lipid A and colistin resistance levels. Strains expressing MCR-1 are also more resistant to antimicrobial peptides and bile acids. Thus, a single residue external to its active site induces ESR activity to confer resilience in MCR-1-expressing strains to commonly encountered environmental stimuli, such as changes in acidity and antimicrobial peptides. Targeted activation of the nonessential protease DegP can lead to the elimination of transferable colistin resistance in Gram-negative bacteria.

**IMPORTANCE** The global presence of transferable *mcr* genes in a wide range of Gram-negative bacteria from clinical, veterinary, food, and aquaculture environments is disconcerting. Its success as a transmissible resistance factor remains enigmatic, because its expression imposes fitness costs and imparts only moderate levels of colistin resistance. Here, we show that MCR-1 triggers regulatory components of the envelope stress response, a system that senses fluctuations in nutrient availability and environmental changes, to promote bacterial survival in low pH environments. We identify a single residue within a highly conserved structural element of *mcr-1* distal to its catalytic site that modulates resistance activity and triggers the ESR. Using mutational analysis, quantitative lipid A profiling and biochemical assays, we determined that growth in low pH environments dramatically increases colistin resistance levels and promotes resistance to bile acids and antimicrobial peptides. We exploited these findings to develop a targeted approach that eliminates *mcr-1* and its plasmid carriers.

## INTRODUCTION

The efficacy of antibiotics in clinical use remains uncertain and is threatened by the looming crisis of widespread antimicrobial resistance (1). Polymyxins, a member of the family of cationic polypeptide antibiotics, are often a final refuge in the treatment of severe infections caused by extremely multidrug-resistant (XDR) Gram-negative pathogens (2). Intrinsic chromosomal resistance of isolates to colistin, a commonly used polymyxin, is nowadays commonplace as a result of mutations in chromosomal genes that either regulate the expression of lipid A-modifying enzymes or modulate the properties of efflux pumps toward the drug (3). The emergence of the transferable mobile colistin resistance gene (*mcr-1*) and its related derivatives has worsened the situation and threatens the revival and continued use of colistin as a last line of defense against pandrug-resistant bacterial infections (4). Additional variants of *mcr-1* on transmissible plasmids continue to be detected, with 10 gene families currently known (*mcr-1* to -*10*) (5). Nevertheless, *mcr-1* remains the most widely disseminated member of this family globally and is found in various species of Gram-negative bacteria isolated from animals, processed meat, vegetable products, and humans (6).

The protein MCR-1 is a membrane-bound enzyme with two domains: an N-terminal-domain with five transmembrane helices (amino acids [aa] 1 to 180) that is buried in the cytoplasmic membrane and a C-terminal domain (aa 219 to 541) with an active site that catalyzes the addition of phosphoethanolamine to lipid A to decrease colistin affinity. Based on homology modeling studies with the full-length *Nm*EptA (the lipid A-modifying phosphoethanolamine [PEA] transferase of Neisseria meningitidis) structure, the loop (aa 181 to 218) connecting these domains is exposed to the periplasm to form a hinge region and bridging helix (7). Presently, crystal structures for only the soluble C-terminal catalytic domain of MCR-1, where both its enzymatic activity and cofactor binding sites reside, are available (8). At least five residues within the active site are critical for the enzymatic hydrolysis of phosphatidylethanolamine, leading to phenotypic colistin resistance (7). Removal of the N-terminal transmembrane domain renders MCR-1 inactive, suggesting that insertion into the cytoplasmic membrane is required for proper juxtapositioning and function of the catalytic domain in the periplasm (9).

The negatively charged lipid A moiety of LPS present in the bacterial outer membrane is the initial target of the cationic antimicrobial peptide colistin. MCR-1 is a membrane-bound Zn^2+^ metalloenzyme that catalyzes phosphoethanolamine transfer onto bacterial lipid A, making bacteria resistant to colistin. This takes place in two reaction steps: the first step, which is rate limiting, involves formation of a covalent phosphointermediate via transfer of PEA from a membrane phospholipid donor to the acceptor Thr285, while in the second step, the phosphoethanolamine group is transferred to the glucosamine phosphate groups of lipid A (9). Thus, MCR-1 exhibits two catalytic activities, that of a phosphatidylethanolamine (PE)-dependent phosphatase and that of a PEA transferase.

Efficient dissemination of antibiotic resistance genes depends on the level of biological fitness imposed on the host strain carrying them (10, 11). Several studies suggest that plasmids bearing *mcr-1* do not impose a fitness cost; however, conflicting results have been reported. Zhang et al. (12) and Nang et al. (13) reported decreased fitness in E. coli and Klebsiella pneumoniae harboring *mcr-1*, respectively. Other reports indicate that plasmid-borne *mcr-1* did not reduce fitness in E. coli but that fitness was impaired in K. pneumoniae (14). In a recent study where isogenic recombinant strains carrying *mcr-1* on the Escherichia coli K-12 chromosome under the control of serial constitutive promoters of different strengths were studied, the fitness costs of tolerable *mcr-1* expression on bacteria were inapparent or low. In particular, no differences in virulence were observed between strains in a mouse model of systemic infection (15). Bacteria carrying cloned versions of *mcr-1* on inducible, high-level-expression plasmids confer resistance to colistin, but this increase in resistance is associated with deleterious effects on growth and fitness loss (12, 16). In separate studies that used transcriptomics and metabolomics to monitor changes in growth following inducible *mcr-1* gene expression in E. coli, it was concluded that fitness adaptation involves PEA modification, pathways in cell envelope biogenesis, iron acquisition, increased lipid metabolism, energy generation pathways and oxidative stress protective mechanisms (15, 17). Nevertheless, the nature and source of the stress induced by bacteria harboring *mcr-1* are currently not known.

Chromosomally encoded colistin resistance mechanisms in *Enterobacterales* are regulated by two-component sensor-regulator systems (TCSs) such as PhoPQ and PmrAB (18). Recently, we demonstrated that PhoPQ TCSs in various Enterobacter species sense changes in the acid composition of growth media to induce high levels of l-Ara4N (4-amino-4-deoxy-l-arabinose)-modified lipid A, thereby conferring colistin resistance (19). PhoPQ, like PmrAB, activate a large set of genes involved in lipopolysaccharide (LPS) modification, efflux pump activities, and numerous other cellular processes, such as regulation of outer membrane integrity and the acid tolerance response (ATR) (18, 20). However, studies examining the expression characteristics of *mcr-1* are largely descriptive and suggest dependence on the type of plasmid that carries *mcr-1* and the strain background of E. coli (21). Thus, little is known of the host regulatory components induced in strains harboring *mcr-1*.

Here, we report that MCR-1 is cleaved by the periplasmic serine endoprotease DegP (22). Mutations at the DegP cleavage site, which is distal to its catalytic site, result in different phenotypes with respect to colistin resistance activity. Production of MCR-1 induces the CpxRA arm of the ESR and affects proton motive force (PMF) maintenance. Its activity is strongly enhanced under conditions of acid stress where MICs of colistin reach high, hitherto-unreported levels.

## RESULTS

### Specific cleavage of MCR-1 by DegP.

**FIG 1:**
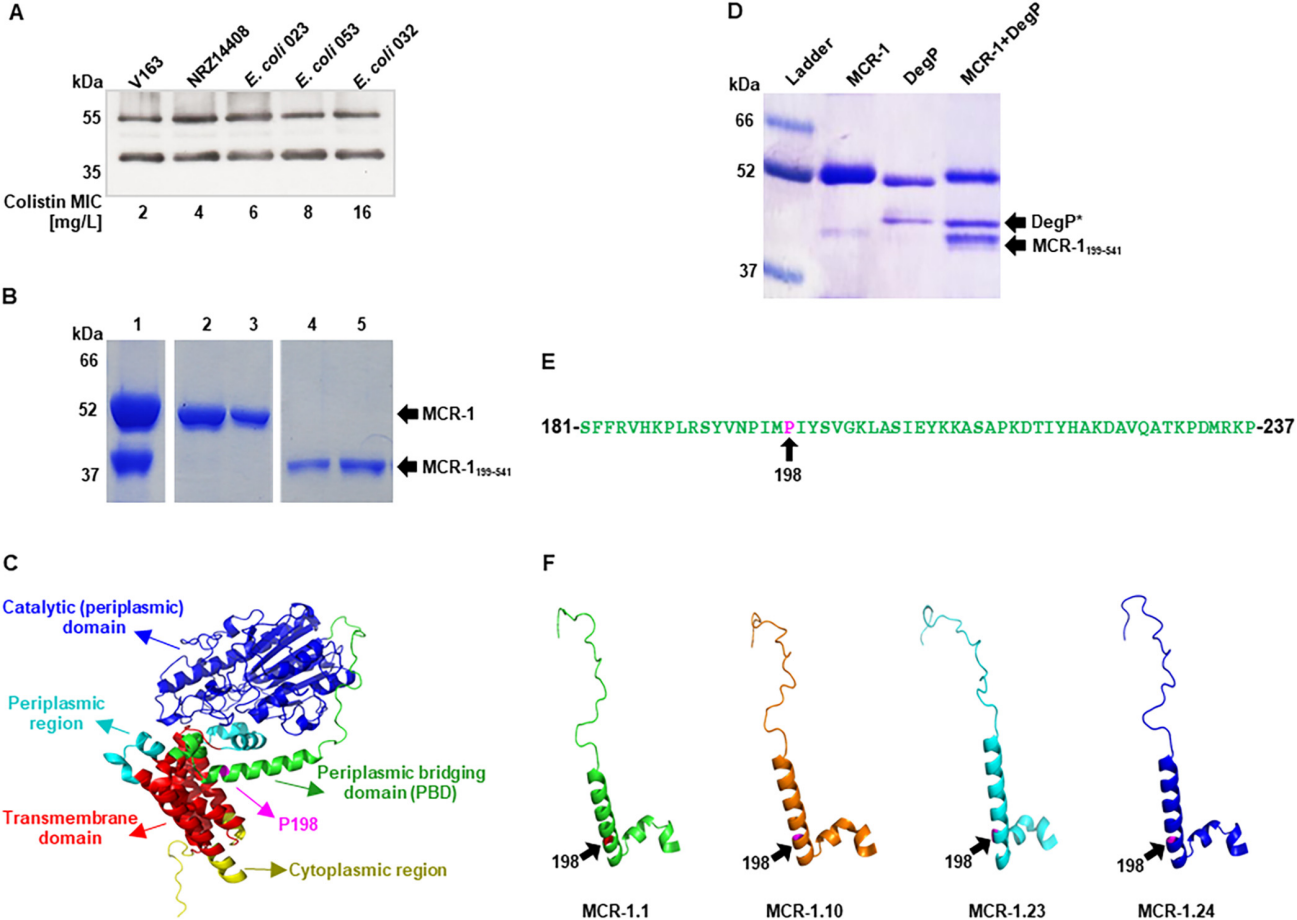
Specific cleavage of MCR-1 by DegP. (A) Immunoblot of MCR-1 in periplasmic fractions of clinical E. coli isolates V163, NRZ14408, E. coli 023, E. coli 053, and E. coli 032 carrying the *mcr-1* gene. Full-length (55 kDa) and cleaved (38 kDa) forms of the protein were detected in all isolates regardless of MICs of colistin. (B) Purification of MCR-1 and detection of a cleaved 38 kDa derivative as observed by Coomassie blue staining following SDS-PAGE. Edman sequencing identified the 38 kDa polypeptide as a fragment of MCR-1 following its cleavage at aa 198. Lane 1, His-tagged purified MCR-1; lanes 2 and 3, MCR-1; lanes 4 and 5, MCR-1_199–541_ following size exclusion chromatography. The full-length protein obtained from this experiment was used for the cleavage studies whose results are presented in panel D and Fig. 2F. (C) Location of the DegP cleavage site within the PBD connecting the N- and C-terminal domains of MCR-1. The crystal structure of MCR-1 was modeled using RoseTTAFold. The different regions of the proteins are labeled and depicted in different colors. (D) Coomassie-stained SDS-PAGE following incubation of purified MCR-1 with DegP. Lane 1, MW ladder; lane 2, MCR-1; lane 3, DegP; lane 4, DegP and MCR-1 coincubated. DegP* is a self-cleavage product of DegP. (E) Amino acid sequence of the PBD (aa 181 to 237) with the DegP cleavage site at aa 198 (in pink). (F) Comparison of the PBD of phylogenomically distant alleles of MCR-1 (see also Fig. S6).

We produced monoclonal antibodies (MAbs) to purified MCR-1 protein and used them to detect the presence of MCR-1 in clinical isolates of E. coli harboring the *mcr-1* gene (23, 24) (see Fig. S1A in the supplemental material). Analysis of periplasmic fractions from various E. coli isolates using SDS-PAGE and subsequent immunoblotting with any one of the anti-MCR-1 MAbs C12, F6, A5, and C4 consistently detected two cross-reacting bands at 55 kDa and 38 kDa (Fig. 1A and Fig. S1B). To understand the origin of this lower-molecular-mass product, we examined protein fractions from our purification scheme for the full-length 55 kDa MCR-1 protein and detected a copurifying 38 kDa polypeptide in several fractions (Fig. 1B and Fig. S1C and D). N-terminus Edman sequencing localized the cleavage site in MCR-1 for the 38 kDa polypeptide within the periplasmic bridging helix, i.e., between its N-terminal transmembrane and soluble periplasmic domains (Fig. 1C and Table S1). The sequence at the cleavage site, PIMP↓IYSV, suggested that it was a substrate of a periplasmic endoprotease. A screen to identify the periplasmic endoprotease involved was performed using a panel of mutants from the Keio collection, where the cleavage of MCR-1 was monitored by immunoblotting (25). These experiments identified DegP as the protease involved (Fig. S1E).

Next, we incubated purified MCR-1 with DegP to confirm that it is indeed the correct protease. SDS-PAGE and Coomassie blue staining revealed the presence of the 38 kDa cleavage product (Fig. 1D). The cleavage site is located within a 57-amino-acid segment (aa 181 to 237) that is highly conserved, in terms of both its amino acid sequence composition and its overall structure, among the different MCR-1 alleles (Fig. 1E and F; Fig. S1F). We designate this structure the periplasmic bridging domain (PBD) of MCR-1. We also examined the ability of the periplasmic chaperones DegQ and SurA to degrade MCR-1. Neither of these proteins exhibited activity toward purified MCR-1 (Fig. S1G and H). Thus, DegP specifically cleaves MCR-1 at aa 198, exposed to the periplasmic space.

### Mutations at the cleavage site have different consequences for colistin resistance activity.

To examine the biological consequences of the periplasmic cleavage site on the activity of MCR-1, we established a noninducible pUC-based system that constitutively expresses wild-type *mcr-1* (see Materials and Methods for MCR-1_WT_ [pMCR-1]) (26). Transformants of the E. coli DH10β strain with the pMCR-1 plasmid exhibited colistin resistance of 4 mg/L, compared to <2 mg/L for the plasmid vehicle pUC19 alone. As the proline residue preceding the cleavage site represents a requirement for DegP-dependent proteolytic activity, site-directed mutagenesis was used to create two derivatives, i.e., converting P↓IYSV to either A↓IYSV (MCR-1_P198A_ [pP198A]) or Y↓IYSV (MCR-1_P198Y_ [pP198Y]). Plasmids encoding variant MCR-1 were transformed into E. coli DH10β, and their activity was assessed by measuring colistin resistance levels. These variants exhibited widely different colistin resistance levels. While the strains expressing MCR-1_WT_ and MCR-1_P198A_ have similar MICs of 4 and 6 mg/L, respectively, the strain harboring the pP198Y plasmid was colistin susceptible, exhibiting an MIC of less than 2 mg/L.

**FIG 2:**
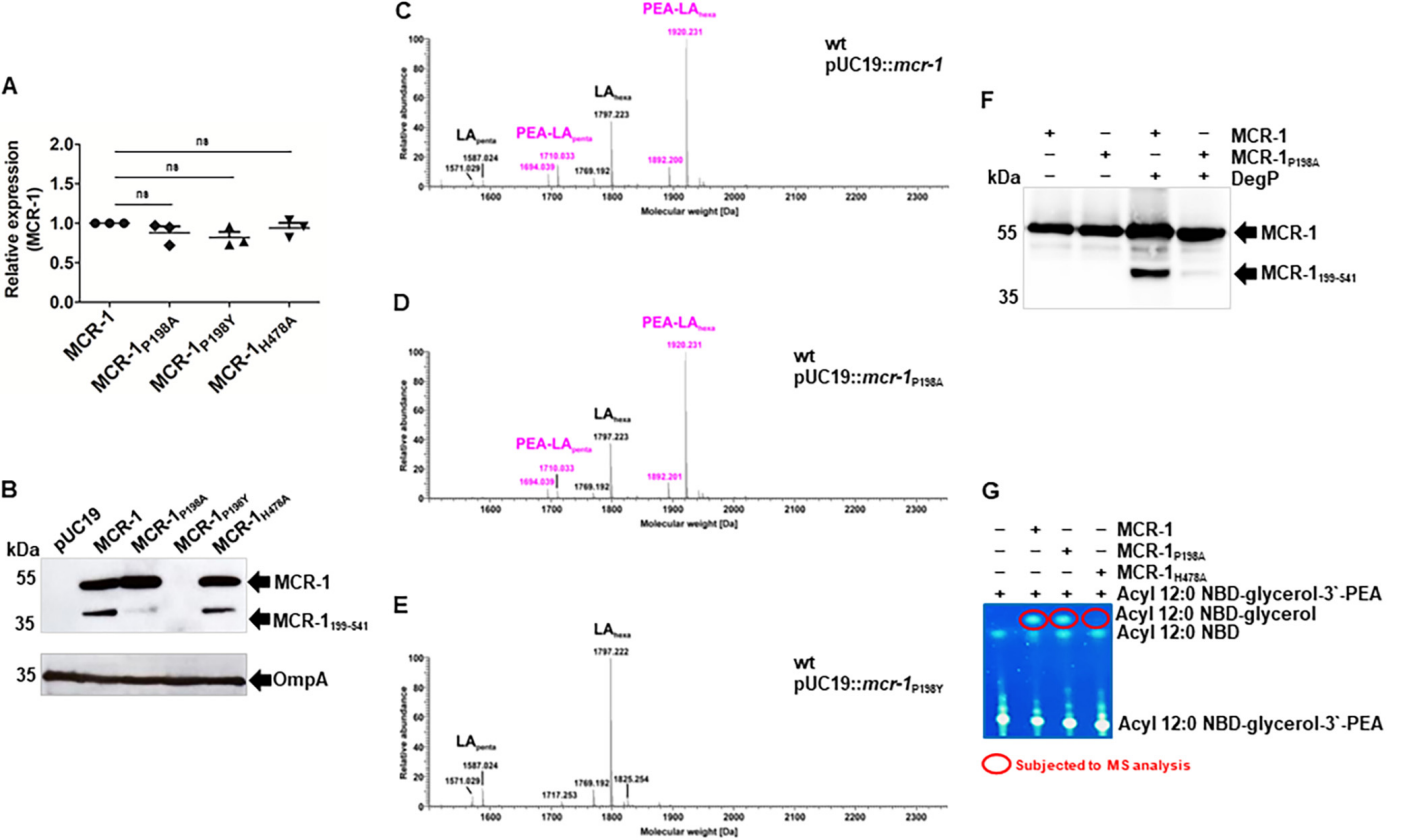
Mutations at the cleavage site have different consequences for colistin resistance activity. (A) qRT-PCR analysis of E. coli DH10β expressing MCR-1_WT_, MCR-1_P198A_, MCR-1_P198Y,_ or MCR-1_H478A._ Data are means and standard errors of the means (SEM) from three independent experiments (ns, not significant). (B) Immunoblots of periplasmic fractions isolated from E. coli DH10β expressing MCR-1 and MCR-1 mutants developed with anti-MCR-1 and anti-OmpA Abs. (C to E) Charge-deconvoluted MS spectra of lipid A preparations for E. coli DH10β strains expressing either MCR-1_WT_, MCR-1_P198A_, or MCR-1_P198Y_ performed in the negative-ion mode and recorded in an *m/z* window of 400 to 2,500. The *m/z* region shown includes penta- to hepta-acylated lipid A species. The typical biphosphorylated, hexa-acylated E. coli lipid A has a calculated mass (*m/z*) of 1,797.219 Da, and the single PEA-modified form has an *m/z* of 1,920.228 Da. Comparable levels of PEA modification of lipid A were detected in MCR-1_WT_ (C) and MCR-1_P198A_ (D) strains but not in the MCR-1_P198Y_ (E) strains. Unmodified lipid A species are indicated with black labels and PEA-modified species with pink labels. Representative spectra of three independent biological replicates of the genotypes are shown. (F) Purified MCR-1_P198A_ is resistant to cleavage by DegP. Immunoblots were developed with anti-MCR-1 MAbs. MCR-1 and its cleavage product are indicated with arrows. (G) The hydrolytic activity of MCR-1_WT_, MCR-1_P198A_, and MCR-1_H478A_ was assessed following overnight incubation of the purified enzymes solubilized in DDM with the proxy fluorescent lipid substrate acyl 12:0 NBD-glycerol-3′-phosphoethanolamine. The cleavage product acyl 12:0 NBD-glycerol was visualized using thin-layer chromatography and verified by MS analysis (see Fig. S2D). Unlike MCR-1_H478A_, which was unable to catalyze PEA hydrolysis, MCR-1_P198A_ catalyzed the hydrolysis of PEA from the lipid substrate, similar to the wild-type enzyme MCR-1.

The copy number of *mcr-1* from cultures of E. coli DH10β strains harboring pMCR-1, pP198A, or pP198Y was assessed by quantitative real-time PCR (qRT-PCR) using 16S rRNA as a standard reference copy number. All isolates showed the same expression level of *mcr-1* (Fig. 2A). Immunoblot analysis of periplasmic protein fractions from the three strains using specific antibodies revealed the polypeptide of 38 kDa in addition to the 55 kDa full-length protein for the strain harboring pMCR-1 (Fig. 2B). The strain producing MCR-1_P198A_ exhibited the 55 kDa full-length protein with a clearly reduced abundance of the 38 kDa cleavage product. For the strain carrying pP198Y, no cross-reacting protein was detected, despite its *mcr-1* expression levels being similar to those of the other two strains, suggesting posttranscriptional protein degradation (Fig. 2B).

We corroborated these data by examining lipid A species from growing cultures using mass spectrometry. Lipid A profiles indicated similar levels of PEA modification of lipid A in bacteria expressing MCR-1_WT_ or the mutant MCR-1_P198A_ (Fig. 2C and D). No PEA modification was detected for the strain carrying the pP198Y plasmid (Fig. 2E). The position of the mono-PEA substitution was determined by tandem mass spectrometry (MS^2^) experiments to be the 4′-phosphate of the lipid A.

We purified MCR-1_P198A_ and confirmed that unlike its wild-type (WT) counterpart, the variant protein is more resistant to degradation by DegP (Fig. 2F and Fig. S2A to C). These results were extended by using an *in vitro* enzymatic assay to assess the removal of the PEA moiety from an alternative substrate, acyl 12:0 NBD-glycerol-3′-phosphoethanolamine (27), by MCR-1 and its derivatives (Fig. 2G and Fig. S2D). For these experiments, we also generated a plasmid expressing MCR-1_H478A_ (see Fig. 2A for transcription profiles and Fig. 2B for protein profiles), which is known to be inactive due to a mutation within the catalytic site, and used purified mutant protein as an assay control (Fig. S2E and F) (28). As expected, substrate hydrolysis, as observed by the release of acyl 12:0 NBD-glycerol, was detected with both the MCR-1_WT_ and MCR-1_P198A_ proteins, while the MCR-1_H478A_ protein lacked this activity (Fig. 2G and Fig. S2D).

### Restoration of MCR-1 expression and activity of the P198Y mutant require ESR components.

**FIG 3:**
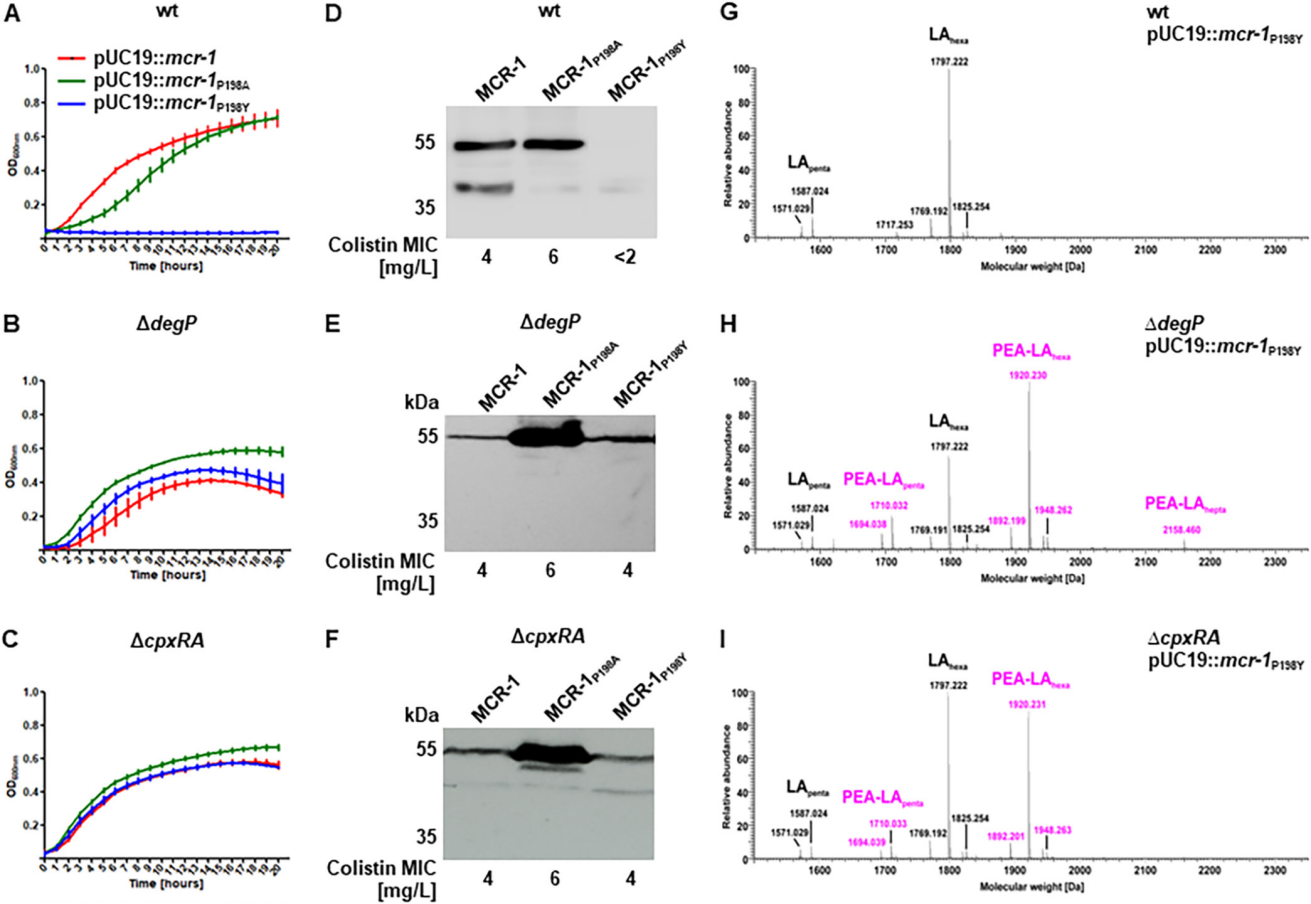
Restoration of MCR-1 expression and activity of the P198Y mutant requires ESR response components. (A to C) Growth curves of E. coli DH10β, DH10β Δ*degP*, and DH10β Δ*cpxRA* expressing MCR-1_WT_, MCR-1_P198A_, or MCR-1_P198Y_ in LB broth supplemented with 2 mg/L colistin. The E. coli DH10β strain harboring the pP198Y plasmid is highly sensitive to colistin (A). Expression of MCR-1_P198Y_ in E. coli DH10β Δ*degP* (B) and E. coli DH10β Δ*cpxRA* (C) restores colistin resistance. Data are means and SEM from three independent experiments. (D to F) Immunoblots of periplasmic fractions isolated from E. coli DH10β, E. coli DH10β Δ*degP*, and E. coli DH10β Δ*cpxRA* expressing MCR-1_WT_, MCR-1_P198A_, or MCR-1_P198Y_. Conversion of the cleavage site from proline to alanine (P198A) renders it resistant to DegP but has no effect on colistin resistance activity. A proline-to-tyrosine (P198Y) mutation of the cleavage site leads to loss of MCR-1 production (D). Expression of MCR-1_P198Y_ is restored in E. coli DH10β Δ*degP* (E) and E. coli DH10β Δ*cpxRA* (F). (G to I) Charge-deconvoluted spectra of the MS analysis of lipid A preparations for E. coli DH10β (G; the image is the same as Fig. 2E and is included for better visualization), E. coli DH10β Δ*degP* (H), and E. coli DH10β Δ*cpxRA* (I) expressing MCR-1_P198Y_ performed in the negative-ion mode and recorded in an *m/z*-window of 400 to 2,500. The region shown covers penta- to hepta-acylated lipid A species. Expression of MCR-1_P198Y_ in E. coli DH10β Δ*degP* (H) or E. coli DH10β Δ*cpxRA* (I) restores PEA modification of lipid A. Unmodified lipid A species are indicated with black labels, and PEA-modified species are shown with pink labels. Representative spectra of three independent biological replicates of the genotypes are shown.

Induction of the periplasmic protease DegP is prominently linked to its regulation by a two-component system made up of the sensor kinase CpxA and its response regulator, CpxR (29). We reasoned that expression of the MCR-1_P198Y_ mutant would be regained in backgrounds lacking the DegP protease. Transformation of the pP198Y plasmid into either an isogenic E. coli DH10β Δ*degP* mutant or an E. coli DH10β Δ*cpxRA* mutant restored the ability to grow in the presence of colistin to levels similar to those seen with both the pMCR-1 and pP198A plasmids (Fig. 3A to C). Immunoblotting experiments confirmed the presence of the MCR-1_P198Y_ protein in these mutants and its absence in the E. coli DH10β parental strain (Fig. 3D to F). We corroborated these findings by examining lipid A profiles from E. coli DH10β and its isogenic Δ*degP* and Δ*cpxRA* mutants, all harboring pP198Y. Comparative mass-spectrometric analysis clearly demonstrated that PEA modification of lipid A is restored when the MCR-1_P198Y_ mutant protein is produced in either E. coli DH10β Δ*degP* or E. coli DH10β Δ*cpxRA* (Fig. 3G to I). A detailed description of all detected lipid A molecular species considered in this study is given in Table S2.

### DegP introduction ameliorates MCR-1-induced stress.

**FIG 4:**
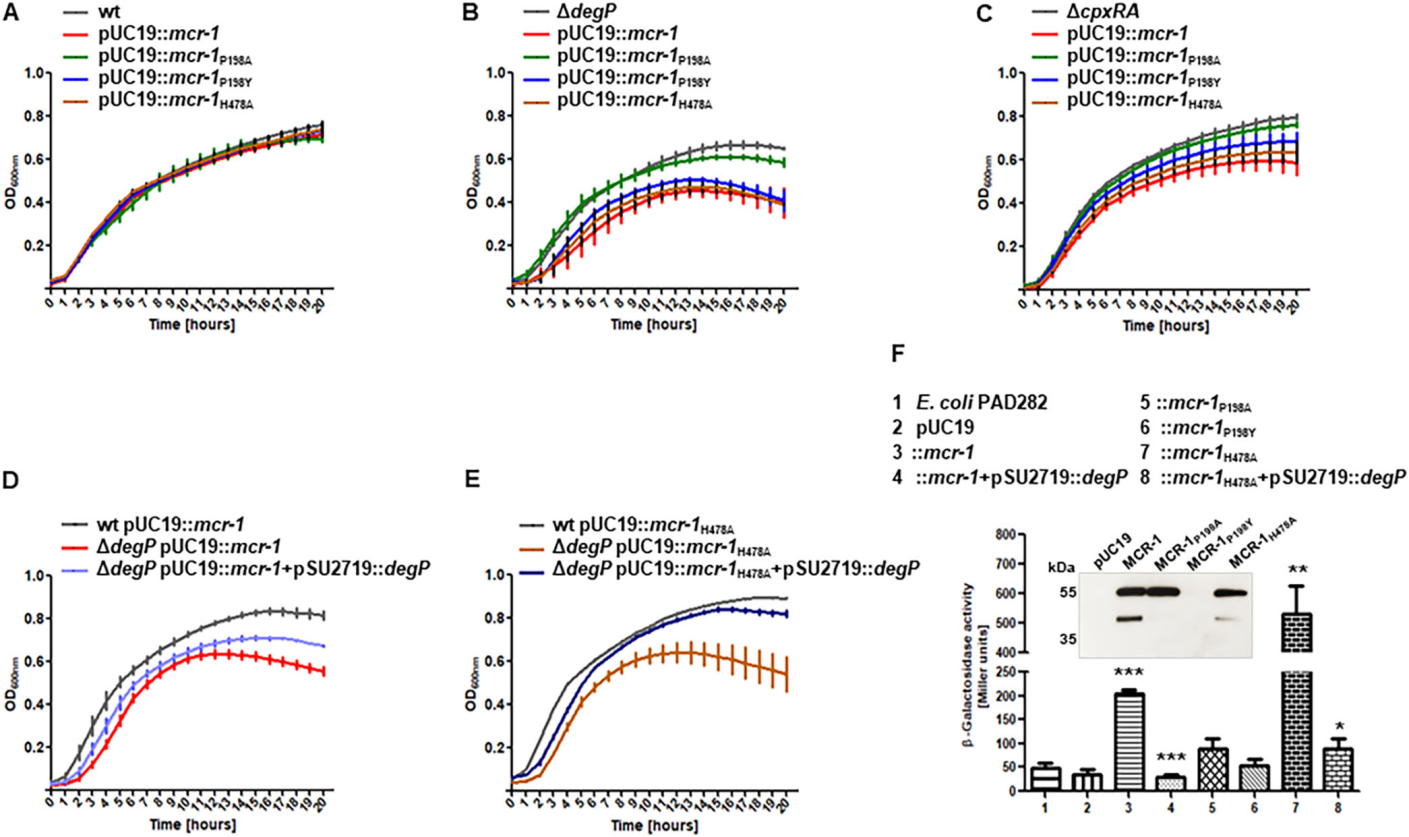
DegP introduction ameliorates MCR-1-induced stress. (A to C) Growth curves of E. coli DH10β (A), E. coli DH10β Δ*degP* (B), and E. coli DH10β Δ*cpxRA* (C) expressing MCR-1_WT_, MCR-1_P198A_, MCR-1_P198Y_, or MCR-1_H478A_ in LB broth supplemented with 100 mg/L ampicillin. MCR-1 expression imposes growth-dependent fitness costs in a Δ*degP* (B) and a Δ*cpxRA* (C) strain. Data are means and SEM from three independent experiments. (D and E) Coexpression of DegP together with MCR-1_WT_ or MCR-1_H478A_ improves growth of E. coli DH10β lacking DegP. Growth suppression induced by MCR-1_WT_ and MCR-1_H478A_ in E. coli DH10β strains lacking DegP (B) or CpxRA (C) is reversed through reintroduction of the *degP* gene. Data are means and SEM from three independent experiments. (F) Induction of *cpxP*::*lacZ* activity in the E. coli PAD282 strain expressing MCR-1_WT_ or MCR-1_H478A_ is DegP dependent. To evaluate induction of the CpxRA pathway, β-galactosidase activity was measured in *cpxP*::*lacZ* reporter E. coli strains expressing WT or mutated MCR-1 variants. The strains expressing MCR-1_WT_ or MCR-1_H478A_ induced high levels of β-galactosidase activity compared to the MCR-1_P198A_- or MCR-1_P198Y_-producing strains. Immunoblots of periplasmic fractions show the same level of protein expression in the MCR-1_WT_-, MCR-1_P198A_-, or MCR-1_H478A_-producing strain. Expression of DegP in the presence of MCR-1_WT,_ or MCR-1_H478A_ alleviated CpxRA pathway-dependent periplasmic stress responses. Data are means and SEM from three independent experiments (*, *P* < 0.05; **, *P* < 0.01; ***, *P* < 0.001).

It has been reported that expression of MCR-1 is tightly controlled, and its production imposes a fitness cost on bacteria harboring it. We examined the growth of E. coli DH10β strains carrying pMCR-1 or its derivatives pP198A, pP198Y, and pH478A in LB medium. No differences in growth were observed for any of the plasmids introduced into this strain (Fig. 4A). Nevertheless, transformation of the same set of plasmids into the isogenic Δ*degP* derivative of E. coli DH10β revealed differences (Fig. 4B). The growth of the strain carrying pP198A was indistinguishable from that of the parental strain E. coli DH10β. The strain expressing MCR-1_P198Y_ exhibited decreased growth. Strains expressing either MCR-1 or inactive MCR-1_H478A_ showed the strongest growth inhibition. A similar result was obtained with the Δ*cpxRA* mutant using this set of plasmids, suggesting that components of the Cpx stress response are involved in the maintenance of plasmids harboring MCR-1 and MCR-1_H478A_ (Fig. 4C).

The Δ*degP* strains harboring either pMCR-1 (E. coli DH10β Δ*degP* pUC19::*mcr-1*) or pH478A (E. coli DH10β Δ*degP* pUC19::*mcr-1*_H478A_) exhibited poor growth characteristics. Reintroduction of the *degP* gene into E. coli DH10β Δ*degP* mutant carrying either plasmid improved bacterial growth, implying a role for the Cpx pathway and, in particular, DegP in managing MCR-1-dependent fitness stress (Fig. 4D and E).

To examine if a general CpxRA-dependent periplasmic stress response was induced in MCR-1-producing strains, we used the E. coli strain PAD282, which carries a reporter *cpxP*::*lacZ* β-galactosidase fusion, to monitor for this activity (30). Following transformation of the various MCR-1 plasmids, recombinant strains were grown in the presence of ampicillin, and the respective β-galactosidase activities were monitored. A strain carrying the pMCR-1 plasmid exhibited 4-fold-higher levels of β-galactosidase activity than strains with the vector plasmid pUC19 or the pP198A and pP198Y plasmids, indicating activation of the Cpx pathway (Fig. 4F). Surprisingly, a 10-fold activation was obtained for a strain carrying the pH478A plasmid expressing an enzymatically inactive MCR-1 protein. These effects were not due to differences in expression levels of the various MCR-1 mutants tested (Fig. 4F; inset). The introduction of the *degP* gene on the medium-copy-number vector pSU2719 (31) suppressed the β-galactosidase activity seen with both pMCR-1 and pH478A (Fig. 4F).

### Growth at acidic pH induces high-level MCR-1-dependent colistin resistance.

**FIG 5:**
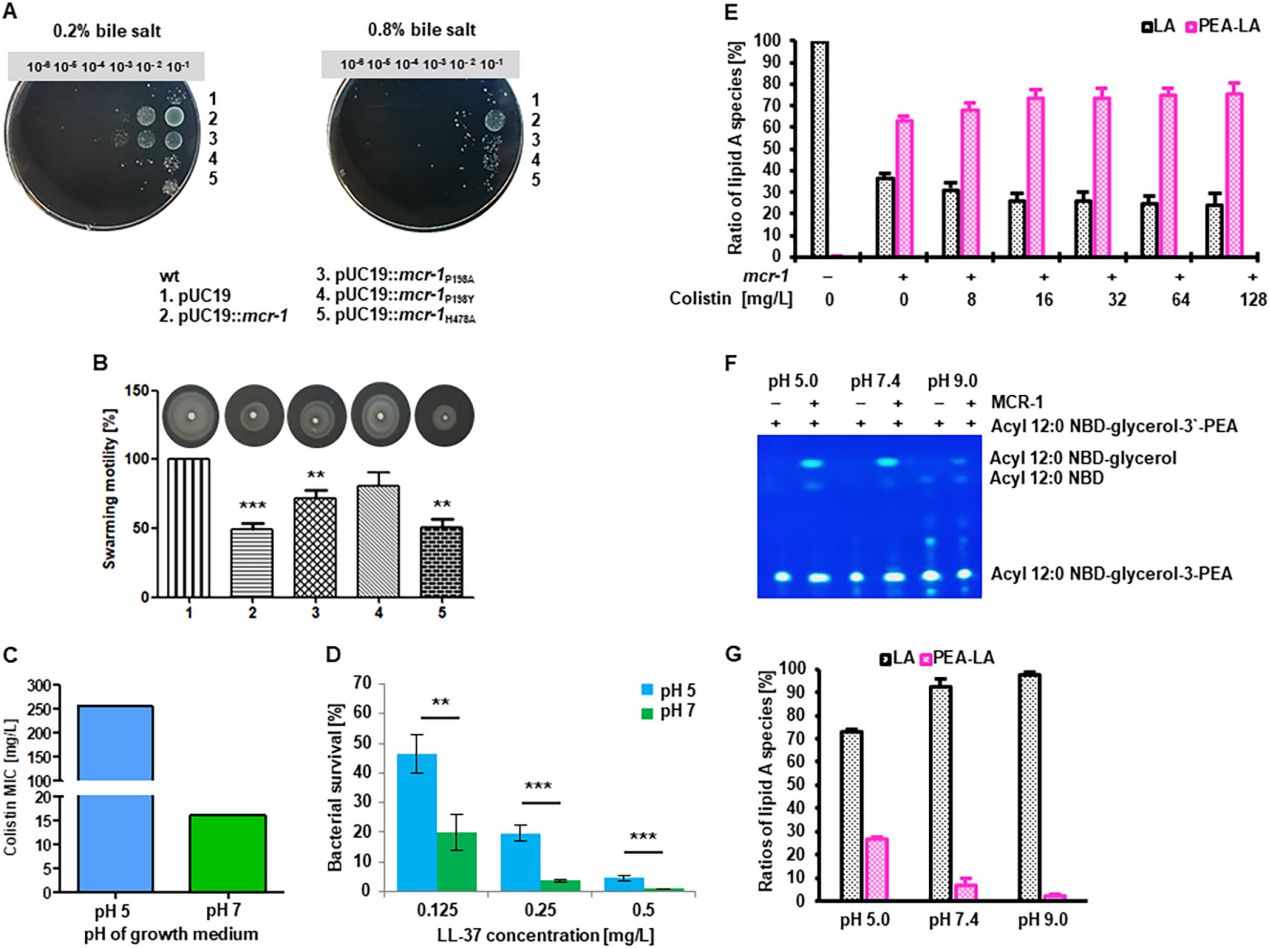
Growth at acidic pH induces high-level MCR-1-dependent colistin resistance. (A) Serial dilutions of E. coli DH10β expressing MCR-1_WT_, MCR-1_P198A_, MCR-1_P198Y_, or MCR-1_H478A_ were spotted directly on the surface of LB agar plates supplemented with 100 mg/L ampicillin and either 0.2% or 0.8% bile salts. Plates were incubated overnight at 37°C. E. coli DH10β strains expressing active MCR-1 (MCR-1_WT_ or MCR-1_P198A_) exhibit bile salt resistance. (B) The motility of E. coli DH10β expressing MCR-1_WT_, MCR-1_P198A_, MCR-1_P198Y_, or MCR-1_H478A_ was assessed on LB agar plates supplemented with 100 mg/L ampicillin and 0.3% agar. Data are means and SEM from three independent experiments (**, *P* < 0.01; ***, *P* < 0.001). MCR-1_WT_- and MCR-1_H478A_-expressing isolates show reduced motility. (C) E. coli DH10β harboring pUC19::*mcr-1* exhibits pH-dependent colistin resistance levels. MIC determination was performed as described in Materials and Methods. Data are representative of three independent experiments. (D) E. coli DH10β harboring pUC19::*mcr-1* exhibits pH-dependent resistance to the antimicrobial peptide LL-37. Data are means and SEM from three independent experiments (**, *P* < 0.01; ***, *P* < 0.001). (E) Ratio of nonmodified (black bars) and PEA-modified (pink bars) lipid A species of E. coli DH10β pUC19 and E. coli DH10β pUC19::*mcr-1* grown at pH 5 in the absence and the indicated concentrations of colistin. Calculation of the ratio of PEA-modified and unmodified lipid A is explained in Materials and Methods and can also be found in Table S2. Exemplary MS spectra are shown in Fig. S4E. (F) The hydrolytic activity of MCR-1 at different pH values was assessed by the incubation of the purified enzyme solubilized in DDM with the fluorescent lipid substrate acyl 12:0 NBD-glycerol-3′-phosphoethanolamine overnight. Incubation was carried out in buffers at different pH values as described in Materials and Methods. The cleavage product acyl 12:0 NBD-glycerol was visualized using thin-layer chromatography. MCR-1-dependent hydrolytic activity is clearly observed at pH 5 and pH 7.4 and is only marginal at pH 9. (G) Ratio of nonmodified (black bars) and PEA-modified (pink bars) lipid A species observed in the *in vitro* reconstitution assay of PEA modification of lipid A by MCR-1 at different pHs. MCR-1 PEA transfer activity is clearly observed at pH 5 and pH 7.4, with a significant increase at acidic pH. Calculation of the ratio of PEA-modified and unmodified lipid A was performed as described in Materials and Methods. Details can be found in Table S2. Exemplary MS spectra are shown in Fig. S5.

Induction of the Cpx response has been previously linked to acid stress adaptation and motility (32). We grew serial dilutions of E. coli expressing MCR-1 and its derivatives on LB plates supplemented with various concentrations of bile salts. Only the strain harboring pMCR-1 grew on plates supplemented with up to 0.8% bile salts (Fig. 5A and Fig. S3A). On plates with 0.2% bile salts, growth of the strains carrying either pP198A or pMCR-1 was comparable, while growth of the pP198Y-, pH478A-, and pUC19-carrying strains was restricted (Fig. 5A and Fig. S3A).

An examination of the recombinant strains on motility agar plates indicated that motility was highly impaired in strains expressing either MCR-1 or its inactive derivative, H478A (Fig. 5B). The strain expressing the P198Y variant was almost as motile as the strain harboring only the pUC19 plasmid, while motility of the strain expressing P198A was somewhat lower.

Previous studies indicate a role for transmembrane potential in both resistance to bile acids and flagellum-based motility (33). To test the hypothesis that MCR-1 activity has effects on the ΔpH component of PMF, we grew bacteria in media at different pH. Lowering the growth medium pH from 7 to 5 dramatically increased *mcr-1*-dependent MIC of colistin from 16 to >256 mg/L (Fig. 5C and Fig. S3B). This effect was not dependent on plasmid copy number, as it was seen with both the pUC plasmid and with the *mcr-1*-carrying IncX4 plasmid pV163M (23). MCR-1-producing bacteria grown at pH 5 were also more resistant to the antimicrobial peptide LL-37, for both pUC and IncX4 plasmids (Fig. 5D and Fig. S3C). To examine if these observations regarding pH-dependent changes in colistin resistance could be generalized, we monitored MICs of colistin among clinical isolates of E. coli (23, 24). We found that only isolates that harbor the *mcr-1* gene, regardless of carrier plasmid type involved, exhibited high levels of resistance to colistin, up to 512 mg/L, when grown in media at pH 5 (Fig. S3D).

We used immunoblotting to examine production of MCR-1 in cultures grown in LB at pH 7 and pH 5. We detected both the 55- and 38-kDa proteins in cultures grown under both conditions even in the absence of colistin. In cultures grown at pH 7, expression of MCR-1 was detectable only up to 8 mg/L of colistin, but in cultures grown at pH 5, expression was detectable even up to 128 mg/L colistin (Fig. S4A to D). Using mass spectrometry we observed a correlation between the proportion of PEA-modified lipid A for bacteria grown at either pH 5 or pH 7 and colistin resistance levels. For bacteria grown at the lower pH, we detected, in addition to an increased proportion of PEA-modified hexa-acylated lipid A, enhanced levels of PEA-modified hepta-acylated lipid A (Fig. 5E; Fig. S4E and Table S2).

### Enzymatic activities of MCR-1 are pH dependent.

The aforementioned results suggested that MCR-1 activities are pH dependent. We studied the purified MCR-1 protein with regard to its pH-dependent enzymatic activities using two assays. We first examined its ability to hydrolyze the synthetic substrate acyl 12:0 NBD-glycerol-3′-phosphoethanolamine at different pH values (Fig. 5F). Hydrolytic activity was detected in enzyme reactions performed at pH 5 and pH 7 but was greatly diminished at pH 9. To demonstrate that MCR-1 also catalyzes the PEA transfer from PE to purified lipid A, we established an *in vitro* reconstitution assay for MCR-1 using LPS isolated from E. coli DH10β and using purified components. Efficiency of transfer was monitored by mass-spectrometric analysis after hydrolytic lipid A release from the LPS. The addition of PEA to lipid A of E. coli DH10β by MCR-1 was strongly pH dependent and was considerably higher at pH 5 than at pH 7 or pH 9 (Fig. 5G; Fig. S5 and Table S2).

### Excipient activation of DegP activity abrogates MCR-1-dependent colistin resistance.

**FIG 6:**
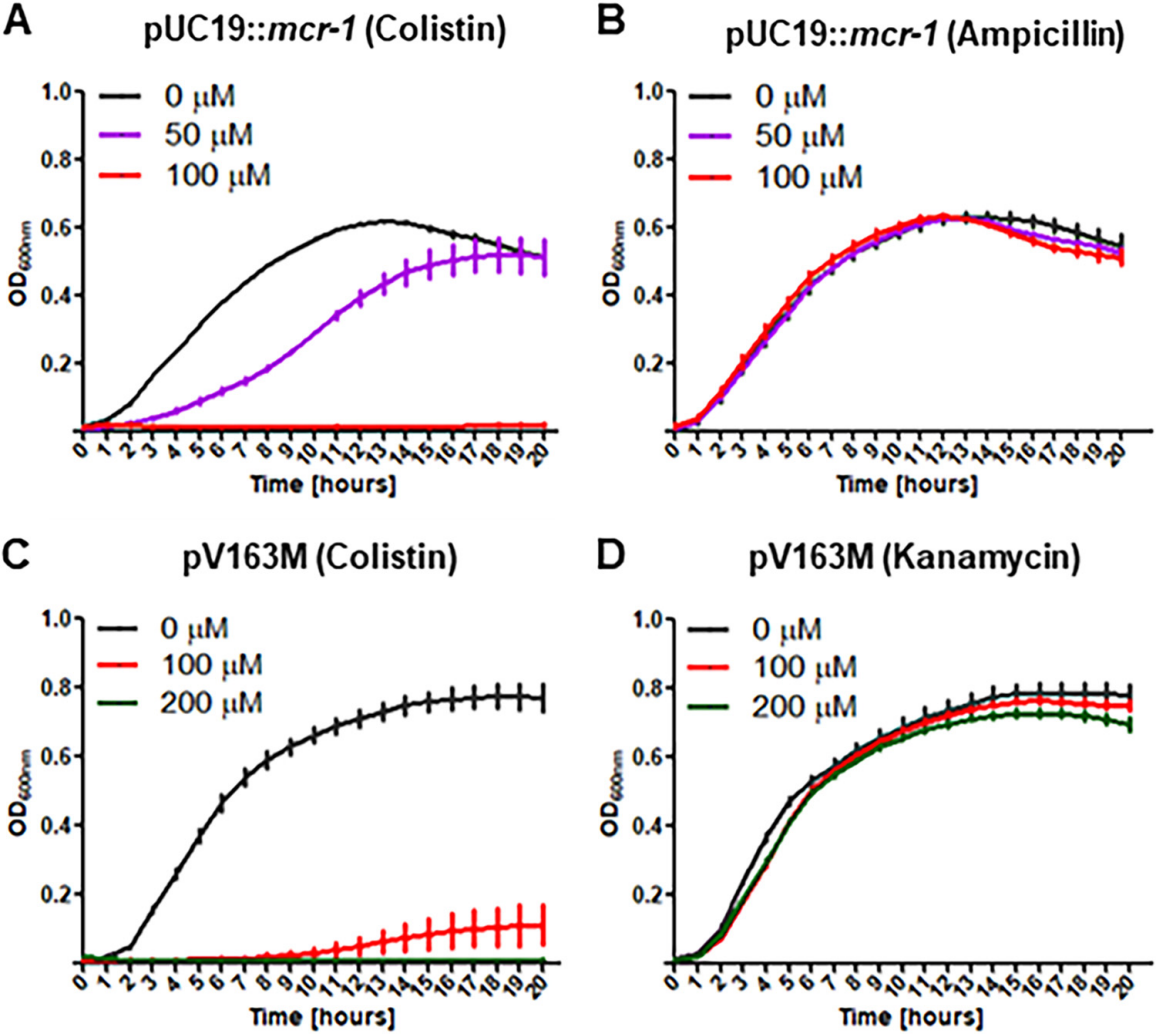
Excipient activation of DegP abrogates growth of strains harboring *mcr-1* plasmids. (A to D) Excipient activation of DegP specifically eliminates strains harboring *mcr-1* plasmids expressing colistin resistance. Loss of colistin resistance for the pUC-based (A) and IncX4-based (pV163M) (C) plasmids detected in the presence of colistin at various concentrations of activator peptides. Experiments were repeated with the same strains using either ampicillin (B) or kanamycin (D); resistance genes for these antibiotics are carried by pUC19 and pV163M, respectively. Here, no growth inhibition is observed. Data are means and SEM from three independent experiments.

The results presented above demonstrate an essential role of the Cpx envelope stress response and its protease component DegP in the control of MCR-1-dependent colistin resistance. We predicted that targeting conformation transition states of MCR-1 through allosteric activation of DegP could be a potential mechanism to eliminate or minimize activity of strains carrying MCR-1. We used a recently described allosteric activator of DegP, the peptide DYFGSALLRV (34), and examined a strain harboring pMCR-1 for loss of the ability to grow in the presence of colistin when exposed to increasing concentrations of the peptide. For these studies, we used an E. coli DH10β Δ*tolC* mutant which improves both uptake and retention of the peptide. As seen in Fig. 6A, increasing concentrations of this peptide in the medium rendered cultures sensitive to colistin, with complete inhibition of growth of bacteria harboring pMCR-1. The effect was specific for *mcr-1*-dependent colistin resistance, because regardless of the concentration of the peptide used, there was no growth inhibition in the presence of ampicillin, a pUC-based selection marker (Fig. 6B). We repeated the experiment using the E. coli DH10β strain carrying the IncX4 plasmid pV163M, which contains both *mcr-1* and an aminoglycoside 3′-phosphotransferase (*aph3*) gene conferring resistance to kanamycin. We found that the activator peptide completely restricted the growth of bacteria carrying the *mcr-1* gene in its original context on the plasmid pV163M only under growth conditions where colistin was selected for (Fig. 6C). Cultures grown in the presence of kanamycin remained unaffected regardless of the peptide concentrations used (Fig. 6D).

## DISCUSSION

The envelope stress response systems of Gram-negative bacteria enable its multilayered envelope, comprising the inner and outer membrane and the peptidoglycan layer within the periplasm, to cope with environmental stresses and is the site for a myriad of functions critical for cellular growth and viability (reviewed in reference 35). As it is an essential component in maintaining cellular homeostasis, its integrity is continuously monitored. In E. coli, this task is accomplished by at least five dedicated ESR systems (Bae, Cpx, Psp, Rcs, and σ^E^), which sense problems in the envelope and conditionally change the transcriptional program to combat the relevant stress (reviewed in reference 36). For example, the Cpx pathway predominantly deals with misfolded inner membrane and periplasmic proteins, and defects in inner membrane protein translocation, peptidoglycan biosynthesis and lipoprotein trafficking, while the σ^E^ pathway senses and responds to defects in the transport and assembly of outer membrane proteins (OMPs) and LPS (32, 37, 38).

The two-component system CpxRA consists of an inner membrane-bound histidine sensor kinase, CpxA, and a cytoplasmic response regulator, CpxR. Depending on the presence of inducing signals, CpxA can act either as a kinase or as a phosphatase on CpxR. The transcription factor CpxR, in its phosphorylated form, regulates expression of *degP* as part of the modulatory responses involving a large number of genes involved in ESR, starvation responses, chemotaxis and motility, and pH tolerance (39). DegP is a periplasmic protease and chaperone that degrades a wide range of substrate proteins in E. coli, many of which are associated with alleviating envelope protein distress (40).

Here, we show that the periplasmic bridging domain (PBD) of MCR-1 is involved in the induction of ESR through activation of the two-component system CpxRA. The induction of an ESR-dependent response activates the periplasmic chaperone/protease DegP, which specifically cleaves MCR-1, and this is reflected in the detection of both the full-length form of MCR-1 at 55 kDa and its C-terminally truncated form of 38 kDa in the clinical isolates tested. Activation of the CpxRA pathway and cleavage by DegP in strains grown at neutral pH is a dynamic process that probably serves to restrict colistin resistance activity, as these molecular forms of MCR-1 are commonly detected in isolates grown in media at physiological pH.

This observation suggests why levels of colistin resistance displayed by MCR-1-producing E. coli are moderate (usually 2 to 8 mg/L ) compared to the level of colistin resistance (usually 8 to 256 mg/L ) mediated by, for example, increased expression of PmrA/PmrB (41). In a study with recombinant strains, engineered to express a chromosomal copy of *mcr-1* in E. coli K-12 under the control of serial constitutive promoters of different strengths, it was observed that despite 200-fold differences in *mcr-1* transcriptional expression, the highest MIC detected was 8 mg/L and comparable to levels obtained for clinical isolates, which had nearly 10-fold-lower *mcr-1* transcription levels (15).

Our studies employed a vector system that allowed production of similar levels of *mcr-1* transcripts from both the wild-type gene and it mutant variants and enabled us to show that the effects observed derive from changes introduced into the protein itself (Fig. 2A to E). We corroborated our findings by examining for changes in phosphoethanolamine modification on lipid A moiety of LPS in mutant strains and developed an *in vitro* assay to assess the ability of purified solubilized MCR-1 and its mutant derivatives to transfer PEA from PE to the lipid A of E. coli (Fig. 2G and Fig. S2D). We found that the activity of the purified MCR-1 is enhanced when assayed at acidic pH (Fig. 5F and G; Fig. S5 and Table S2). Notably, it has been reported that under the acidic conditions in which MCR-1 activity is enhanced, DegP completely loses its protease activity (42).

Support for a role of ESR following MCR-1 expression was obtained by Feng et al. (43), who suspected a link between MCR-1 and ESR based on studies examining damage to outer membrane permeability. Specifically, the study showed that the MCR-1 production increased expression of genes controlled by Cpx (e.g., *degP*, *degS*, *nlpE*, and *cpxP*), Rcs (e.g., *rcsF*, *rseP*, and *rcsD*), Bae (e.g., *baeR* and *baeS*), and Psp (e.g., *pspF* and *pspA*) (43). Other studies have also indicated an interaction between the CpxRA-regulated periplasmic dithiol-disulfide oxidoreductase DsbA and MCR-1 (44).

Generally, the acid limit for E. coli growth is around pH 4.0 to 4.5, and currently known acid resistance (AR) and acid tolerance (ATR) systems only prolong survival of E. coli cells under acidic conditions but cannot support growth at pH 4.0 to 4.5 (45). Recently, it was shown that the two-component system CpxRA enables E. coli to directly sense acidification through protonation of the CpxA periplasmic histidine residues. This enhanced transcription of the essential genes *fabA* and *fabB* required for biosynthesis of unsaturated fatty acids (UFAs) leads to an increase of UFA content in membrane lipid, thus enabling E. coli to grow at acidic pH (20). This newly described CpxRA-dependent ATR would, for example, favor growth of MCR-1-expressing bacteria in media containing bile acids (Fig. 5A and Fig. S3A).

We exploited these observations, *viz.*, that CpxRA promotes growth of MCR-1-expressing bacteria at moderately low pH and that DegP activity is reduced at low pH to assess the MICs of MCR-1 strains grown in low pH media. We observed dramatic increases of up to 512 mg/L for all the *mcr-1*-expressing E. coli strains tested, regardless of strain background or plasmid type, suggesting that this adaptive mechanism is highly conserved in these bacteria. Embedding *mcr-1*-dependent resistance activity within the ESR would promote anticipatory resilience in strains within host tissue environments, such as during passage through the orogastrointestinal tract or when bacteria are endocytosed into the acidic lysosomal subcellular compartments of host cells.

As cleavage of MCR-1 by DegP is a defining characteristic, we performed comparative analysis of its cleavage site at position 198 (PIMP↓IYSV) in proteins belonging to the *mcr* gene family (Fig. S6A). This revealed that this cleavage site is invariant and highly conserved only in MCR-1, not in other members of this family of proteins. Permissive mutations at this site, such as those described here with P198A, which neither induce an ESR nor lead to a loss of colistin resistance activity were not detected. However, it may be that they exist but are either unfavorable and confer no benefits or are simply rare and have not been detected.

Recently, a similar PBD, designated linker 59, was detected in MCR-3 and implicated as a facilitator of colistin resistance (46). The amino acid sequences making up linker 59 are very different from those found in the PBD structure of MCR-1, but they share strong overall structural similarity (Fig. S6B). Linker 59 appears to facilitate colistin resistance activity, but the mechanism underlying this observation is not known. Comparative sequence analysis of MCR-like enzymes suggests that these PBDs are specific to the different classes of MCR family of proteins and may have evolved as a result of domestication of a particular lipid A-PEA transferase (46), possibly adapted to species-specific ESR components (Fig. S6 and S7). This would reflect observations from studies suggesting that these enzymes have evolved from phylogenetically distant bacterial species, such as *Moraxella* spp. for MCR-1 and *Aeromonas* spp. for MCR-3 (47).

Our results suggest that targeting the conformational stability of MCR-1 rather than its activity could be an effective strategy to eliminate bacteria harboring *mcr-1*. The periplasmic heat shock protease DegP is carefully regulated and controlled by allosteric activation (34, 48), and targeted excipient enhancement of DegP protease activity would be expected to abrogate colistin resistance. Allosteric activation of DegP resulted in growth inhibition of strains producing MCR-1 in the presence of colistin, an effect not seen when cultures were grown in the presence of other antibiotics, such as ampicillin or kanamycin; resistance genes that are also present on the plasmids tested. Thus, targeting superactivation of the DegP and related Htr proteases is a promising approach to control the spread of plasmids harboring the transmissible *mcr-1* gene worldwide.

**FIG 7:**
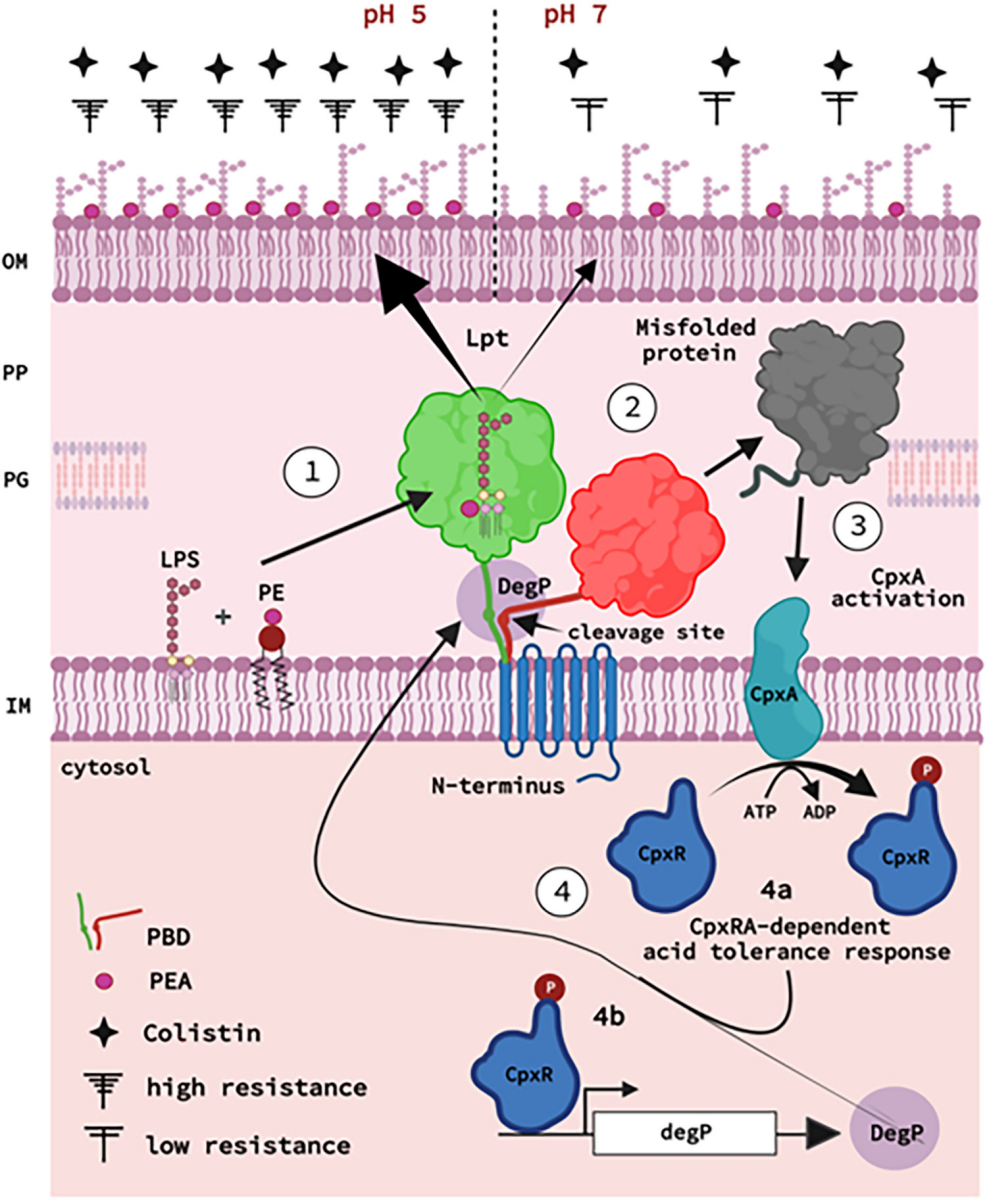
Model of MCR-1-dependent activation of CpxRA processes. MCR-1 is represented with its inner membrane (IM) N-terminal transmembrane region (TM; blue), its periplasmic bridge region (PBD; green, active; red, inactive), and its C-terminal catalytic domain (CD; green, active; red, inactive). DegP (brown) and its cleavage site on MCR-1 at aa 198 are indicated. The enzyme MCR-1 hydrolyzes the substrate phosphatidylethanolamine (PE) and transfers phosphoethanolamine (PEA) to lipid A (1). During growth at neutral pH, both active and inactive (cleaved) forms of MCR-1 are present (step 2) (Fig. S4A and C). At low pH, only the active MCR-1 form predominates (Fig. S4B and D), and its activity is also enhanced (Fig. 5E to G; Fig. S4E and S5), favoring PEA modification of LPS (1). Accessibility to the conserved cleavage site activates the ESR-sensing two-component system CpxRA (3) to induce an acid-tolerant response, enabling MCR-1-producing bacteria to grow in bile salts (step 4a) (Fig. 5A), and promotes the expression of DegP, leading to the enhanced cleavage of MCR-1 (step 4b) (Fig. 1 to 4). (Core sugars and fatty acids of the LPS are only drawn symbolically and do not reflect exact structures).

In conclusion, we report the unique effect of a single residue within the highly conserved PBD structural element in MCR-1 that modulates its enzymatic activity and confers acquired growth-selective advantages to the strains harboring *mcr-1* (Fig. 7). The lack of a three-dimensional structure for the full-length enzyme is a significant impediment to a deeper understanding of the mechanistic basis of the structural features described here. We found that structural bridges linking the different domains of lipid A-PEA transferases are widespread across the order *Enterobacterales* and may have evolved as preemptive motifs to link resistance activity and resilience responses. The identification of MCR-1 as a DegP substrate will also encourage studies on the CpxRA-DegP pathway in other members of the *Enterobacterales*, particularly with respect to the similarities in the architecture of the ESR pathway, as it can now be explored through introduction of a single gene and selection of a simple phenotype imparting colistin resistance. Indeed, given the adaptive mechanism described here, acquiring MCR-1 would appear to be a highly efficient mechanism for microbes to establish protection against universally occurring changes in environmental pH and antimicrobial peptide-based anti-infectives. Alternative pharmaceutical interventions based on the protease-activating strategy described here could lead to the development of new classes of antibiotics that target its dissemination and loss.

## MATERIALS AND METHODS

### Strains, plasmids, and growth conditions.

The bacterial isolates and recombinant strains used in this study can be found in Table S3. Primers are also listed in Table S3. The E. coli V163 isolate harboring the *mcr-1*-carrying IncX4 plasmid pV163M was used as the source of the *mcr-1* gene. The E. coli MG1655 strain was used as the source of the *degP* gene. E. coli DH5α, E. coli DH10β, and E. coli BL21(DE3) were used for cloning and protein expression, respectively.

Unless otherwise stated, all bacteria were grown at 37°C and 180 rpm in liquid Luria-Bertani (LB) medium supplemented with 50 mg/L kanamycin, 100 mg/L ampicillin, 20 mg/L chloramphenicol, or different concentrations of colistin sulfate.

### Cloning of *mcr-1* and *degP*.

For cloning of *mcr-1* in pUC19, *mcr-1* was amplified with Q5 high-fidelity DNA polymerase using the primers *mcr-1*_pUC19_F and *mcr-1*_pUC19_R and inserted into pUC19 after double restriction digestion with the restriction enzymes SalI and EcoRI. The primers were designed to create a so-called ATGA construct. In this construct, the ATG of *mcr-1* gene and the TGA (stop codon) of the preceding *lacZ* gene fragment overlap. Such an operon structure was first detected in the *ninR* region of bacteriophage lambda and subsequently used as an expression system for target genes (26).

For protein purification, the *mcr-1* gene was amplified with Q5 high-fidelity DNA polymerase using the primers listed in Table S3 and cloned into pET-28a(+) by Gibson assembly (49). The *mcr-1* gene in pET-28a(+) contains a C-terminal hexa-histidine (His) tag. The cloning of *degP* gene into the pSU2719 vector was carried out using the same cloning strategy and the primers listed in Table S3.

### Mutagenesis of *mcr-1* gene in pUC19 and pET-28a(+).

The P198A, P198Y, and H478A substitutions in the *mcr-1* gene of pUC19::*mcr-1* and pET-28a(+)::*mcr-1* were introduced using Q5 site-directed mutagenesis according to the manufacturer instructions. The sequence of the oligonucleotides carrying desired substitutions are shown in Table S3. For verification of the mutated gene, whole-genome DNA was isolated using the PureLink genomic DNA minikit following the manufacturer’s instructions. A Nextera XT library of the genome was sequenced with a MiSeq system (using 2 × 300 cycles). Raw data were assembled using SPAdes, and comparison with the wild-type *mcr-1* gene was performed using BLASTn.

### Deletion of *degP* and *cpxRA* genes in E. coli DH10β.

Deletion mutants of E. coli DH10β were generated by homologous recombination using the λ-Red recombination system (50). Briefly, the vector pSIM5-tet (51) carrying the λ-Red genes (γ, β, and *exo*) was introduced into E. coli DH10β. Next, a kanamycin resistance cassette was generated by PCR using the pKD4 vector that carries the kanamycin resistance gene flanked by flippase (FLP) recognition target (FRT) sites. The primers used for the generation of the PCR product included at the 5′-end 40-nucleotide (nt) extensions that are homologous to regions adjacent to the *degP* or *cpxRA* genes and, at the 3′ end, 20-nt priming sequences for pKD4 (Table S3).

Transformants carrying the λ-Red genes were grown in LB medium containing tetracycline (6 mg/L) at 30°C to an optical density at 600 nm (OD_600_) of 0.4. After thermal induction of λ-Red genes (15 min at 42°C), transformants were washed three times with ice-cold 10% glycerol to make them electrocompetent. Following electroporation of the PCR product, deletion mutants were selected on LB agar plates containing 50 mg/L kanamycin at 30°C. The vector pSIM5-tet was eliminated by growing the deletion mutants at 42°C.

The kanamycin resistance cassette was eliminated by using the helper plasmid pCP20, an ampicillin- and chloramphenicol-resistant plasmid that shows temperature-sensitive replication and thermal induction of the FLP recombinase. Kanamycin-resistant mutants were first transformed with pCP20 and then selected on ampicillin agar plates at 30°C. After incubation at 42°C, mutants were tested for loss of all antibiotic resistances. Gene deletions were confirmed by whole-genome sequencing.

### RNA extraction, cDNA synthesis, and qRT-PCR.

Overnight cultures of E. coli DH10β pUC19, pUC19::*mcr-1*, pUC19::*mcr-1*_P198A_, pUC19::*mcr-1*_P198Y_, and pUC19::*mcr-1*_H478A_ were diluted 1:50 in fresh LB medium supplemented with 100 mg/L ampicillin. Afterward, bacteria were grown to an OD_600_ of 1. Aliquots of 1 mL were mixed with RNAprotect reagent and pelleted by centrifugation (9,000 × *g*, 5 min at 4°C). The RNA was isolated using the miRNeasy minikit according to the manufacturer’s protocol. Synthesis of cDNA from RNA was performed via reverse transcription using SuperScript II reverse transcriptase and random hexamer and nonamer primers. The QuantiTect SYBR green PCR kit was used for cDNA amplification of *mcr-1* and the reference 16S rRNA gene (primers are listed in Table S3) using the StepOnePlus real-time PCR system (Applied Biosystems, Darmstadt, Germany). The specificity of the amplicons was confirmed by melting curves and agarose gel electrophoresis. The experiments were done in three biological replicates. Relative quantification was calculated using the mathematical model described by Pfaffl (52).

### Purification of MCR-1.

A starter inoculum was prepared by adding a single colony of E. coli BL21(DE3) expressing a C-terminally His-tagged MCR-1 to 20 mL LB medium containing 50 mg/L kanamycin and growing it overnight at 37°C at 180 rpm. The overnight culture was subcultured in 1 L of fresh LB medium and incubated at 37°C and 180 rpm until the OD_600_ reached 0.6. MCR-1 expression was induced by the addition of isopropyl-β-d-thiogalactopyranoside (IPTG) to a final concentration of 1 mM. At 3 h postinduction, cells were harvested at 8,671 × *g* for 30 min at 4°C. The pelleted cells were resuspended in phosphate-buffered saline (PBS) supplemented with a protease inhibitor mix. Lysis was performed using a Mixer Mill MM 400 (Retsch GmbH, Haan, Germany). The lysate was then sonicated with a B12 Sonifier cell disruptor (Branson Ultrasonics Corporation, Brookfield, CT, USA) and centrifuged at 17,000 × *g* for 30 min at 4°C. The pellet was resuspended in 10 mL of 20 mM sodium phosphate (pH 7.4), 500 mM NaCl, 5 mM imidazole, and 1% DDM (dodecyl β-d-maltoside). The mixture was then incubated at 4°C for 4 to 12 h with end-over-end rotation and subsequently centrifuged at 17,000 × *g* for 60 min at 4°C. The resulting supernatant containing the MCR-1 solubilized in DDM detergent micelles was filtered using a 0.22-μm syringe filter (Merck Millipore, Darmstadt, Germany) and applied to a HisTrap HP 5-mL affinity chromatography column (GE Healthcare, Chicago, IL, USA) equilibrated with binding buffer (20 mM sodium phosphate [pH 7.4], 500 mM NaCl, 5 mM imidazole, and 0.023% DDM) using an ÅKTA purifier fast protein liquid chromatography (FPLC) system (GE Healthcare, Chicago, IL, USA). The unbound proteins were eluted from the column with binding buffer until the absorbance at 280 nm reached a stable baseline value. The bound MCR-1 was eluted from the column using an increasing linear gradient of elution buffer (20 mM sodium phosphate [pH 7.4], 500 mM NaCl, 500 mM imidazole, 0.023% DDM). A peak corresponding to MCR-1 eluted between 40% and 50% elution buffer volume. SDS-PAGE was performed to assess the purity of the eluted peak fractions. Fractions of pure protein were pooled and concentrated, and the buffer was changed to PBS containing 0.023% DDM using a 10 kDa-molecular-weight-cutoff (MWCO) centrifugal filter unit (Amicon Ultra-15, Ultracel 10K; Merck KGaA, Darmstadt, Germany) to 5 mg/mL as determined by the Bradford protein assay.

### Purification of MCR-1_P198A_ and MCR-1_H478A_.

The MCR-1 mutants MCR-1_P198A_ and MCR-1_H478A_ were purified as described for MCR-1_WT_. Additional steps included size exclusion chromatography for MCR-1 and MCR-1_H478A_ and anion-exchange liquid chromatography for MCR-1_P198A._

### Size exclusion chromatography.

Pooled fractions from the HisTrap HP 5-mL affinity chromatography column containing MCR-1 were concentrated with Vivaspin 6, 5000 MWCO PES (Sartorius AG, Gottingen, Germany) to 2 mL and separated using HiLoad 16/60 Superdex 75 prep-grade columns (Cytiva Europe GmbH, Freiburg, Germany) at a flow rate of 1 mL/min. Afterward, the fractions were analyzed by 10% SDS-PAGE followed by Coomassie blue staining.

### Anion-exchange liquid chromatography.

Pooled fractions from the HisTrap HP 5-mL affinity chromatography column were applied to an anion-exchange liquid chromatography column (MonoQ 5/50 GL; Cytiva Europe GmbH, Freiburg, Germany) with a flow rate of 0.5 mL/min using a binding buffer composed of 20 mM Tris-HCl (pH 8.0) and 0.023% DDM. After washing the column with binding buffer to remove nonbound material, the protein was eluted using a linear gradient of 20 column volumes of elution buffer (20 mM Tris-HCl, 1 M NaCl, 0.023% DDM). The fractions were analyzed by 10% SDS-PAGE followed by Coomassie blue staining. Fractions containing the protein were pooled and rebuffered in PBS (pH 7.4) with 0.023% DDM.

### Purification of DegP, DegQ, and SurA.

Purification of DegP (22), DegQ (53), and SurA (54) has been previously reported.

### Mouse monoclonal antibody production.

Mouse monoclonal antibodies (C12, F6, A5, and C4) against MCR-1 protein were produced via an immunization and hybridoma development approach with a commercial partner, Eurogentec (Liège, Belgium). The purified monoclonal antibodies were stored at −20°C.

### V8 protease digestion and monoclonal antibody screen.

Five hundred micrograms of purified MCR-1 was digested with 5 μg of V8 protease for 16 h at 37°C. Afterward, the cleavage products were separated by 15% SDS-PAGE and semidry transferred to a polyvinylidene difluoride (PVDF) membrane for 1 h at 125 V, 54 mA, and 100 W. Next, the membrane was blocked with 5% skim milk in Tris-buffered saline supplemented with Tween20 (TBST) for 1 h. After three washing steps (5 min each) with TBST, the membrane was cut into strips and incubated with supernatant from monoclonal cell lines diluted 1:10 in TBST. The membrane strips were washed again three times for 5 min with TBST and incubated with horseradish peroxidase (HRP)-conjugated anti-mouse antibody diluted 1:2,000 in TBST for 1 h.

### Determination of the DegP cleavage site.

The cleavage site of DegP within MCR-1 was determined through N-terminal sequencing after Edman degradation of the cleavage product (details can be found in Table S1). For this, eluted fractions from the size exclusion chromatography were separated by 10% SDS-PAGE and semidry transferred to a PVDF membrane. After staining of the transferred proteins with amido black, the cleavage product was cut out for sequencing via N-terminal Edman degradation performed on a Procise protein sequencer 494C (Applied Biosystems, Foster City, CA, USA).

### Proteolytic assays.

Proteolytic assays were performed by incubation of DegP (1.35 μM) with either MCR-1 (3.4 μM) or MCR-1_P198A_ (3.5 μM) in PBS supplemented with 0.023% DDM for 4 h at 42°C. The reactions were stopped by boiling the samples in SDS loading buffer at 95°C for 10 min. Afterward, the samples were analyzed by 8% SDS-PAGE followed by Coomassie blue staining. The proteolytic assay performed with purified SurA (1.33 μM) was carried out as described for DegP. The DegQ (1.15 μM) proteolytic assay was performed at pH 5.5 in MES [2-(*N*-morpholino)-ethanesulfonic acid] buffer supplemented with 0.023% DDM.

### Extraction of periplasmic protein fraction by cold osmotic shock.

The periplasmic protein fraction was isolated from freshly harvested bacterial cells by using a cold osmotic shock method (55). Overnight cultures were diluted 1:100 in fresh LB medium and incubated at 37°C and 180 rpm to an OD_600_ of 1.0. Afterward, 5-mL bacterial cultures were pelleted and resuspended in ice-cold cell fractioning buffer 1 (0.2 M Tris-HCl, 200 g/L sucrose, 0.1 M EDTA) at one-fourth of the former suspension volume. Samples were incubated for 20 min on ice and inverted at regular intervals to counteract sedimentation. Following centrifugation for 15 min at 14,000 × *g* and 4°C, the supernatant was removed and the pellet was resuspended in ice-cold cell fractioning buffer 2 (0.01 M Tris-HCl [pH 8.0], 0.005 M MgSO_4_, 0.2% SDS, 1% Triton X-100) at one-fourth of the former suspension volume. Samples were incubated again for 20 min on ice under regular inversion. The samples were recentrifuged for 15 min at 14,000 × *g* and 4°C, and the resulting supernatant was stored at −20°C. The protein concentration was measured using a bicinchoninic acid (BCA) assay.

### SDS-PAGE and immunoblotting.

Following electrophoresis, proteins were visualized by Coomassie blue staining. The protein gels were stained overnight with a mixture of 10% acetic acid, 50% methanol, and 40% H_2_O with the addition of 0.25% (wt/vol) Coomassie brilliant blue R-250 at room temperature on a shaker. Afterward, the protein gels were destained using a mixture of 10% acetic acid, 40% methanol, and 50% H_2_O on a shaker by replacing with fresh rinse mixture until the excess dye has been removed.

Periplasmic protein fractions were separated by 15% SDS-PAGE and semidry transferred to PVDF membranes for 1 h at 125 V, 54 mA, and 100 W. Following protein transfer membranes were blocked with 5% skim milk in TBST for 1 h. Membranes were washed three times for 5 min with TBST. Afterward, MCR-1 and derivatives were detected using mouse monoclonal antibodies diluted 1:20 in TBST containing 5% skim milk. One hour later, the membranes were washed again three times for 5 min with TBST. Next, the membranes were incubated for 1 h with the corresponding anti-mouse antibody conjugated with HRP diluted 1:2,000 in TBST containing 5% skim milk. After three washing steps (5 min each) with TBST the proteins were detected using an Agfa Curix 60 instrument (Agfa-Gevaert AG, Mortsel, Belgium).

### Bile acid assays.

Overnight cultures were diluted 1:100 in fresh LB medium and incubated at 37°C and 180 rpm to an OD_600_ of 1.0. Subsequently, 5-μL serial dilutions of E. coli DH10β expressing MCR-1, MCR-1_P198A_, MCR-1_P198Y_, or MCR-1_H478A_ as well as the control strain containing the empty vector pUC19 were spotted directly on the surface of LB agar plates supplemented with 100 mg/L ampicillin and different concentrations of bile salts. Plates were incubated overnight at 37°C.

### Motility assays.

Overnight cultures of E. coli DH10β expressing MCR-1_WT_, MCR-1_P198A_, MCR-1_P198Y_, or MCR-1_H478A_ as well as the control strain carrying the empty vector pUC19 were diluted 1:100 in fresh LB medium and incubated at 37°C and 180 rpm to an OD_600_ of 1.0. Afterward, 5 μL of bacterial cultures was spotted directly on the surface of LB agar plates supplemented with 100 mg/L ampicillin and 0.3% agar. The plates were incubated overnight at 37°C. The swarming motility of bacterial strains was determined by measuring the diameter of the zone of spreading (56). Experiments were carried out in triplicate.

### Growth kinetics.

Overnight cultures were diluted 1:50 in fresh LB medium and then inoculated into a 96-well microtiter plate. Bacterial growth was recorded by monitoring OD_600_ every 20 min for 20 h at 37°C using the Tecan Infinite M200 Pro microplate reader (Tecan Group Ltd., Männedorf, Switzerland). Using the data analysis software Magellan for plate readers, the raw OD_600_ data were exported into a Microsoft Office Excel sheet with the addition of kinetic time stamps and temperature values. Experiments were repeated in three separate assays.

### Antimicrobial susceptibility testing.

MICs of colistin were assayed by the broth microdilution method (BMD) in cation-adjusted Mueller-Hinton Broth (CA-MHB) as recommended by the European Committee on Antimicrobial Susceptibility Testing (EUCAST [https://www.eucast.org/clinical_breakpoints/]). Briefly, overnight cultures were diluted 1:100 in fresh LB medium and grown until the OD_600_ reached 1.0. These cultures were diluted again 1:100 into a 96-well microtiter plate (200 μL per well) in CA-MHB containing various concentrations of colistin ranging from 0 to 512 mg/L. The plates were incubated overnight at 37°C, and the optical density was measured using a Tecan Infinite M200 Pro instrument. Experiments were repeated three times.

### Cationic antimicrobial peptide LL-37 assay.

Approximately 1,000 CFU of mid-logarithmic-phase E. coli DH10β pUC19::*mcr-1* and E. coli DH10β pV163M cultivated in LB medium at pH 5 and 7 were incubated with 0.06, 0.125, 0.25, and 0.5 mg/L LL-37 in a 96-well microtiter plate at 37°C for 2 h in 200 μL PBS supplemented with 1% LB broth. Afterward, samples were plated onto LB agar plates to evaluate the surviving bacteria by colony counting. The assay was run in triplicates for each peptide concentration.

### Beta-galactosidase activity assays.

Bacterial cultures were grown overnight and then diluted 1:100 in fresh LB medium. Afterward, the bacterial cultures were grown until the mid-exponential phase was reached, with an OD_600_ of 0.5. Cell pellets were washed once with PBS and resuspended in Z-buffer (57). The OD_600_ of cell suspensions was measured using a Tecan Infinite M200 Pro instrument. Cell suspensions were further diluted, and the cells were permeabilized by chloroform-SDS treatment. After a short incubation, the samples were transferred to a 96-well microtiter plate, and the reaction was started by addition of *o*-nitrophenyl-β-d-galactopyranoside (ONPG). Absorbance at 420 nm and 550 nm was measured with the Tecan Infinite M200 Pro instrument. The data collected were analyzed using a Microsoft Excel spreadsheet, and the β-galactosidase activity was calculated in Miller units.

### NBD-glycerol-PEA TLC-based assay.

The hydrolytic activity of MCR-1 was determined by using a substrate containing a fluorescent label, 1-acyl-2-{12-[(7-nitro-2-1,3-benzoxadiazol-4-yl)amino]dodecanoyl}-*sn*-glycero-3-phosphoethanolamine (27) (acyl 12:0 NBD-glycerol-3′-PEA). To evaluate hydrolysis of the PEA group from the substrate catalyzed by MCR-1, 2 μg of purified enzyme was added to 2 μg of lipid substrate and incubated for 20 h at room temperature. The reaction mixtures were used in thin-layer chromatography (TLC) on silica gel 60 plates, and reactions were developed using ethyl acetate-methanol-water (7:2:1 [vol/vol/vol]). The fluorescence signal on the plate was visualized with the Molecular Imager Gel Doc XR system (Bio-Rad Laboratories GmbH, Feldkirchen, Germany). For confirmation of product formation (1-acyl-2-{12-[(7-nitro-2-1,3-benzoxadiazol-4-yl)amino]dodecanoyl}-*sn*-glycerol), the appropriate material was scraped off the TLC plate and eluted in a mixture of chloroform and methanol (3:1 [vol/vol]). The concentrated eluent was analyzed by mass spectrometry.

### LPS extraction.

LPS was extracted from E. coli DH10β by the hot-phenol-water method as described previously with some modifications (58). In brief, bacteria were grown at 37°C under shaking (180 rpm) in LB broth until the OD_600_ reached 1. Afterward, bacteria were harvested by centrifugation (10 min at 6,238 × *g* and 4°C) and washed three times with H_2_O. Pellets were then resuspended in H_2_O and sonicated on ice. Next, an equal volume of hot (65°C) phenol was added to the mixture followed by vigorous shaking at 65°C for 15 min in a water bath. Suspensions were cooled on ice and centrifuged for 30 min at 6,238 × *g* and 4°C. The aqueous phase was transferred to a fresh Greiner centrifuge tube and re-extracted with an equal volume of hot (65°C) H_2_O. Residual phenol in the aqueous phase was removed by extensive dialysis against water at 4°C using Spectra/Por 3 dialysis membranes (MWCO, 3,500) (Avantor, Inc., Radnor, PA, USA). The extracted LPS was lyophilized with a Lyovac GT2 instrument (Leybold Heraeus, Cologne, Germany) and stored at −80°C.

### Lipid A isolation from whole cells.

Lipid A was extracted from freshly harvested bacterial cells using the isobutyric acid-ammonium hydroxide method (59). Briefly, overnight cultures were diluted 1:100 into fresh LB broth and incubated at 37°C and 180 rpm until they reached an OD_600_ of 0.8. Then, 5-mL bacterial cultures were pelleted and washed once with PBS. The pellets were resuspended in 400 μL of 70% isobutyric acid and 1 M ammonium hydroxide (5:3 [vol/vol]) and incubated for 2 h at 100°C. The mixtures were cooled on ice and centrifuged at 2,000 × *g* and 4°C for 15 min. The resultant supernatants were diluted with water (1:1 [vol/vol]) and lyophilized with Lyovac GT2. The samples were then washed twice with methanol and centrifuged at 2,000 × *g* and 4°C for 15 min. The insoluble lipid A fraction was solubilized and extracted with 100 μL of a mixture of chloroform, methanol, and water (3:1.5:0.25 [vol/vol/vol]). After centrifugation at 2,000 × *g* and 4°C for 5 min, the supernatant was transferred to dark glass vials and dried under a hood.

### *In vitro* reconstitution assay of MCR-1 activity.

Purified and lyophilized LPS (2 mg/mL) from E. coli DH10β was resuspended in 300 μL MES buffer (pH 5), PBS (pH 7.4) or sodium borate buffer (pH 9) supplemented with 0.023% DDM. Afterward, 2 mg/mL phosphatidylethanolamine (PE), the donor of phosphoethanolamine (PEA), and 8.3 μg/mL purified MCR-1 were added to the LPS. The reaction mixtures were incubated at 37°C for 24 h with end-over-end rotation. The lipid A was liberated from LPS by the isobutyric acid-ammonium hydroxide method as described above.

### Mass spectrometry.

All mass-spectrometric analyses were performed on a Q Exactive Plus spectrometer (Thermo Fisher Scientific, Bremen, Germany) using a Triversa Nanomate platform (Advion, Ithaca, NY, USA) as the nano-electrospray ionization (ESI) source. Lipid A extracts were initially dissolved in 20 or 25 μL chloroform-methanol-water (60:30:5 [vol/vol/vol]) or chloroform-methanol (8:2 [vol/vol]). Five microliters of this solution was mixed with 95 μL of water–propan-2-ol–7 M triethylamine–acetic acid (50:50:0.06:0.02 [vol/vol/vol/vol]). In cases where the resulting solutions were too viscous for spraying, 1:10 dilutions in water–propan-2-ol–7 M triethylamine–acetic acid (50:50:0.06:0.02 [vol/vol/vol/vol]) were used for measurements. All samples were centrifuged briefly prior application to the MS. Mass spectra were recorded for 0.50 min in the negative mode in an *m/z* range of 400 to 2,500 applying a spray voltage set to −1.1 kV. All depicted mass spectra were charge deconvoluted (Xtract module of the Xcalibur 3.1 software [Thermo Fisher Scientific, Bremen, Germany]) and given mass values referring to the monoisotopic masses of the neutral molecules, if not indicated otherwise. For the calculation of the ratios of nonmodified and PEA-modified lipid A species, mass spectrometric data of 39 acquisitions, including 3 blanks, were imported into one database using LipidXplorer 1.2.8 (60). Monoisotopic peaks of 50 lipid A species were calculated and assigned. Intensities of all observed tetra- to hepta-acylated lipid A species were considered and utilized to determine substitution ratios. Details on the lipid A molecular species can be found in Table S2. MS^2^ experiments for further detailed analysis of the lipid A structure were performed as described previously (61). All samples of the NBD-glycerol-PEA TLC-based assay were diluted 20-fold in the spray solution comprising water–propan-2-ol–7 M triethylamine–acetic acid (50:50:0.06:0.02 [vol/vol/vol/vol]) and then centrifuged briefly at 16,000 × *g* at room temperature. To improve spray stability, 20 μL was carefully transferred to a new vial containing 40 μL of the spray solution, and the mixture was again centrifuged briefly before mass spectrometric data acquisition.

### *In silico* modeling and phylogenomic analysis.

Visualization and annotation of the genome were carried out using Unipro UGENE v39.0 (62). The amino acid sequence alignment and determination of the number of differences in the amino acids and subsequent phylogeny were carried out in Mega v10.8 (63). Transmembrane helices in proteins were predicted using TMHMM 2.0 (https://services.healthtech.dtu.dk/service.php?TMHMM-2.0). MCR protein models were created using RoseTTAFold (64). The stereochemical qualities of modeled structures of MCR were evaluated using a Ramachandran plot (https://www.umassmed.edu/zlab/software/). The MCR models were visualized and annotated in PyMOL v1.7.4.5. Sequence similarities and secondary structure information from aligned sequences were calculated by ESPript3 (65).

### Statistical analysis.

Statistical analyses for all experiments were performed using GraphPad Prism (GraphPad Prism version 5.01 for Windows; GraphPad Software, San Diego, CA, USA [www.graphpad.com]) and Microsoft Excel 2018 (Microsoft Corporation [https://office.microsoft.com/excel]). Results are presented as means from three independent replicates. Student’s *t* test was used to determine the significance of the differences observed between compared groups.

### Data availability.

All the data supporting the findings of this study are available within the article and its supplemental material files. All mentioned data are also available from the corresponding author upon request.
