# Supplementary material for: A Single Residue within the MCR-1 Protein Confers Anticipatory Resilience
Source: Microbiol Spectr. 2023 Apr 18;11(3):e03592-22. doi: 10.1128/spectrum.03592-22 (PMC10269488; doi:10.1128/spectrum.03592-22)
Supplement: Supplemental file 1 — Supplemental material. Download spectrum.03592-22-s0001.pdf, PDF file, 4.4 MB [file spectrum.03592-22-s0001.pdf]

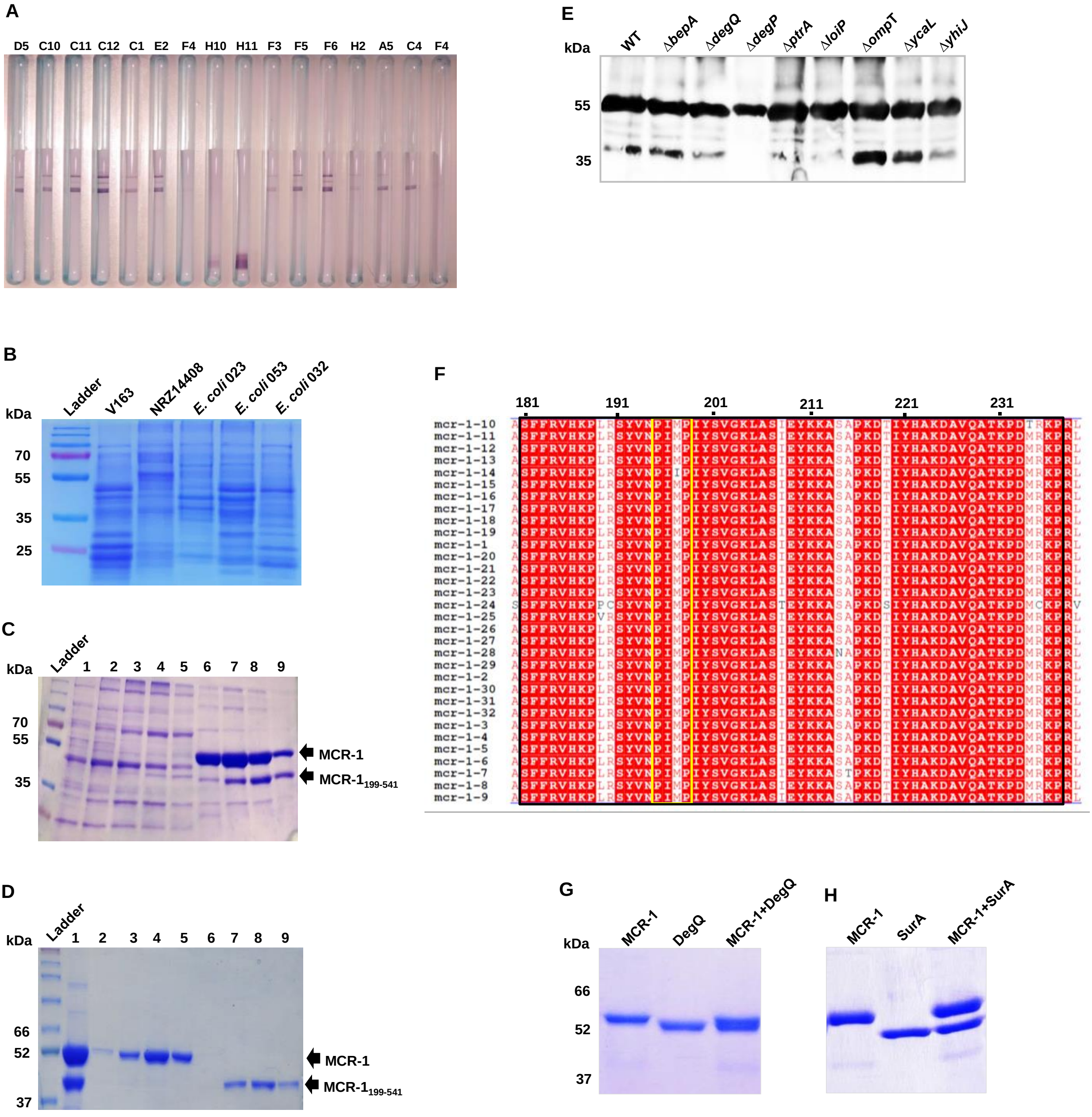

FIG S1 Specific cleavage of MCR-1 by DegP. (A) Monoclonal antibody screen. (B) Coomassie-stained SDS-PAGE of periplasmic fractions of clinical *E. coli* isolates. (C) Coomassie-stained SDS-PAGE of MCR-1 purified by His-tag affinity chromatography. (D) Coomassie-stained SDS-PAGE of MCR-1 following size-exclusion chromatography. Source of data for Fig. 1B. (E) Immunoblot analysis of periplasmic fractions of selected protease mutants from 'Keio collection' expressing MCR-1. (F) Multiple sequence alignments of the PBD of MCR-1 alleles. A comparison of amino acids at region 181-237 of different MCR-1 alleles revealed, except for MCR-1.14, the 'PIMP' region is extremly conserved. (G and H) Coomassie-stained SDS-PAGE following incubation of purified MCR-1 with either DegQ (G) or SurA (H).

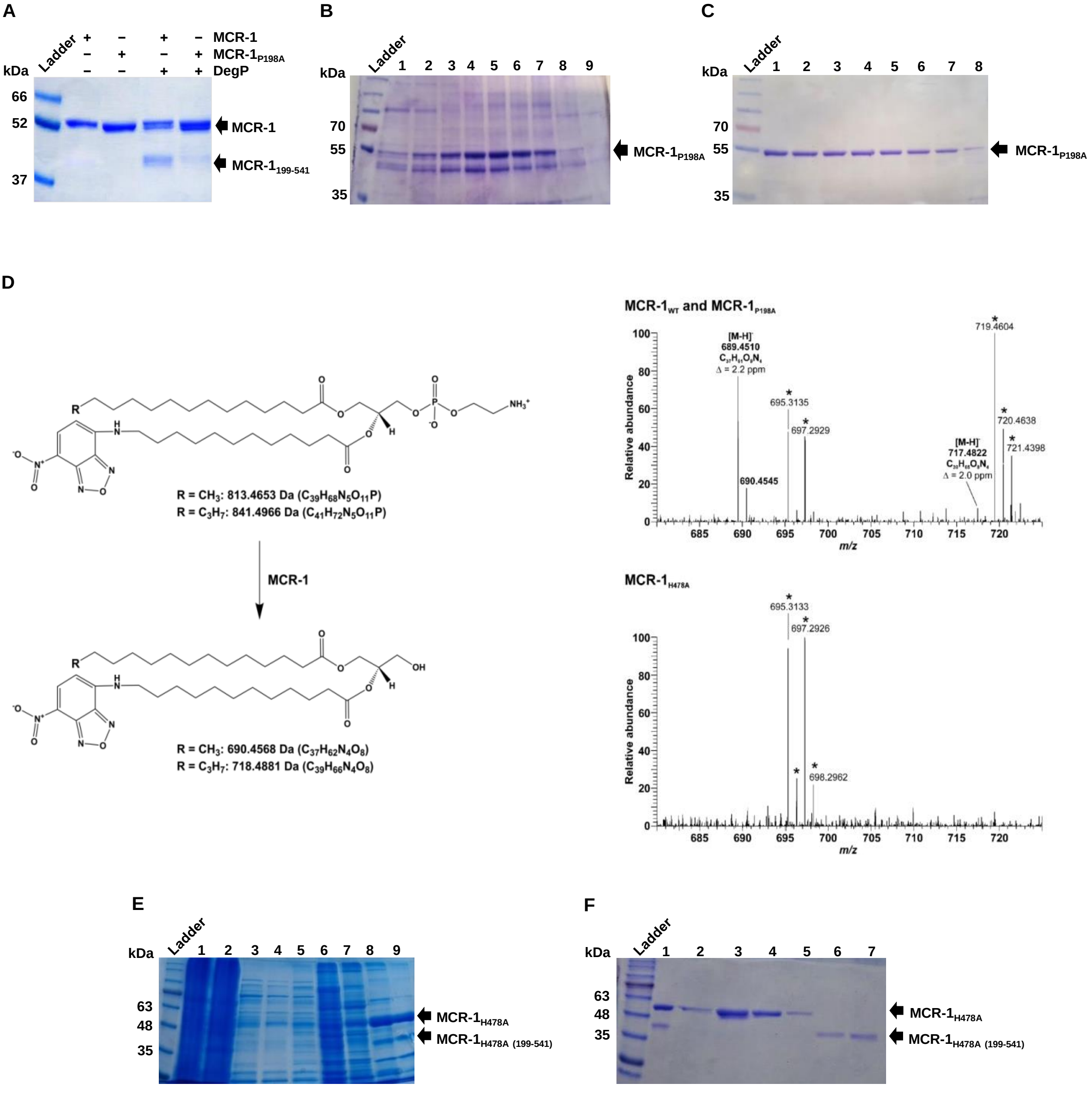

FIG S2 Mutations at the cleavage site have different consequences for colistin-resistance activity. (A) Coomassie-stained SDS-PAGE of the co-incubation of DegP with either MCR-1, or MCR-1_{P198A}. (B and C) Purification of MCR-1_{P198A}. Coomassie-stained SDS-PAGE of MCR-1_{P198A} purified by His-tag affinity chromatography (B). Coomassie-stained SDS-PAGE of MCR-1_{P198A} following anion-exchange liquid-chromatography (C). (D) Mass spectra of products formed by the reaction of acyl 12:0 NBD glycerol-3-PEA with MCR-1_{WT}, MCR-1_{P198A}, or MCR-1_{H478A} (TLC shown in Fig. 2G). Only MCR-1_{WT} (spectrum shown) and MCR-1_{P198A} exhibit the cleavage product 1-acyl-2-{12-[7-nitro-2-1,3-benzoxadiazol-4yl)amino]dodecanoyl}-*sn*-glycerol. Signals marked with * are background signals. (E and F) Purification of MCR-1_{H478A}. Coomassie-stained SDS-PAGE of MCR-1_{H478A} purified by His-tag affinity chromatography (E). Coomassie-stained SDS-PAGE of MCR-1_{H478A} following size-exclusion chromatography (F).

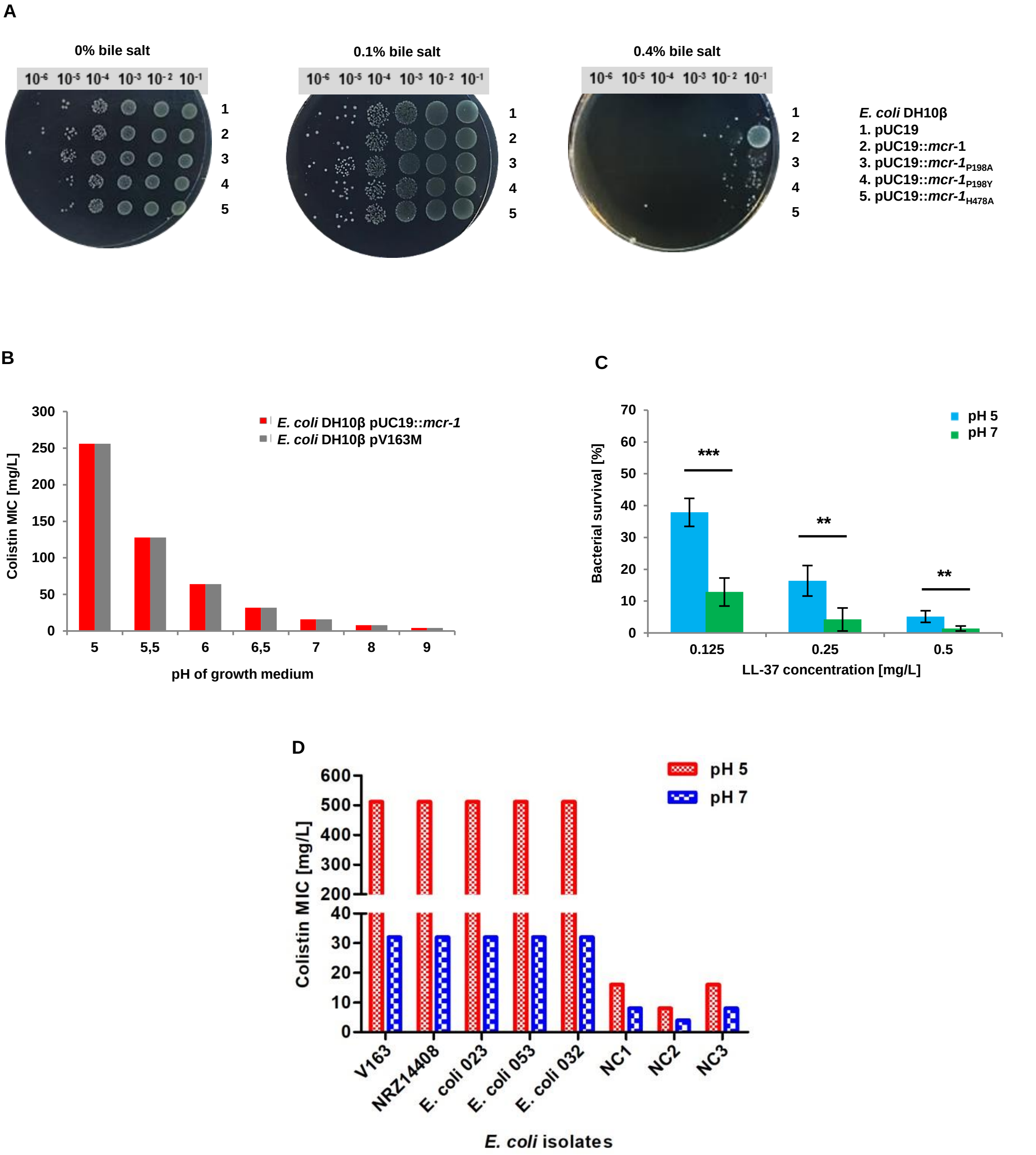

FIG S3 Media dependent expression of high-level MCR-1-dependent colistin resistance. (A) Bile salt resistance assays. Growth on 100 mg/L ampicillin plates without and with 0.1 and 0.4% bile salt are depicted. (B) pH-dependent MICs of *E. coli* DH10β pUC19::*mcr-1* and *E coli* DH10β pV163M. (C) *E. coli* DH10β harboring the IncX4 plasmid pV163M exhibits enhanced resistance to the antimicrobial peptide LL-37 at pH 5. (D) Clinical isolates of *E. coli* harboring *mcr-1* exhibit media pH-dependent MICs towards colistin. The isolates V163, NRZ14408, *E. coli* 023, *E. coli* 053, and *E. coli* 032 all harbor *mcr-1*, while isolates NC1 (SurvCare 230), NC2 (SurvCare 221), and NC3 (SurvCare254) do not carry the *mcr-1* gene.

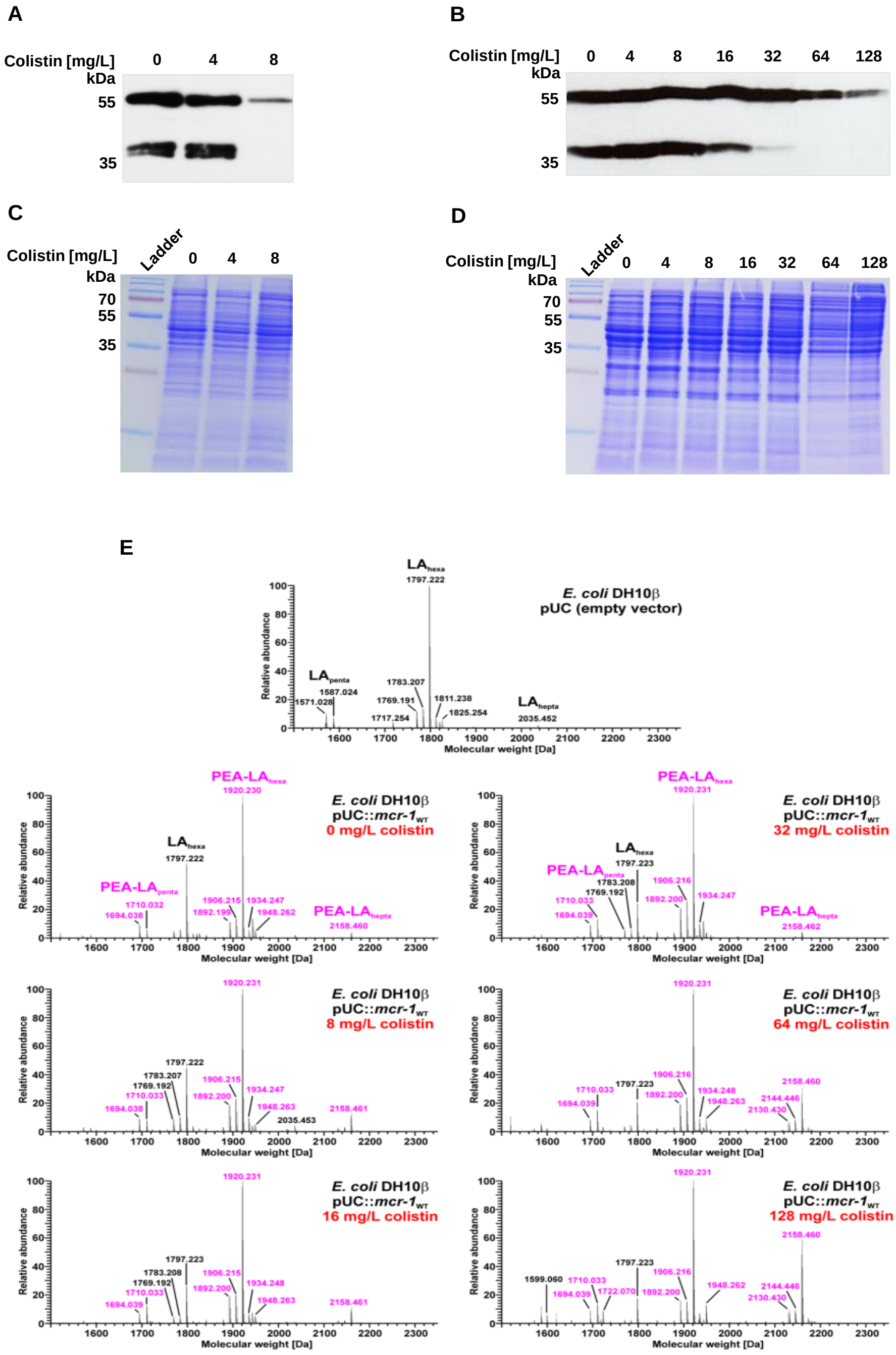

FIG S4 High level of MCR-1-dependent PEA-modification of lipid A at acidic pH. (A to D) MCR-1 expression profiles in cultures grown at pH 7 and pH 5 in the presence of increasing colistin concentrations. Immunoblots of periplasmic fractions isolated from *E. coli* DH10β expressing MCR-1_{WT} grown at either pH 7 (A) or pH 5 (B) are shown. Coomassie-stained SDS-PAGE of periplasmic fractions of *E. coli* DH10β isolates harboring pMCR-1_{WT} obtained from cultures grown at either pH 7 (C) or pH 5 (D). (E) Charge-deconvoluted MS spectra of lipid A preparations of *E. coli* DH10β pUC19 and *E. coli* DH10β pUC19::*mcr-1* cultures grown at pH 5 in absence and indicated concentrations of colistin. The presented *m/z*-region includes penta- to hepta-acylated lipid A species. Unmodified lipid A species are labelled in black, PEA-modified species in pink. Results shown are representative of three (pUC19) or four (pUC19::*mcr-1*) independent biological replicates.

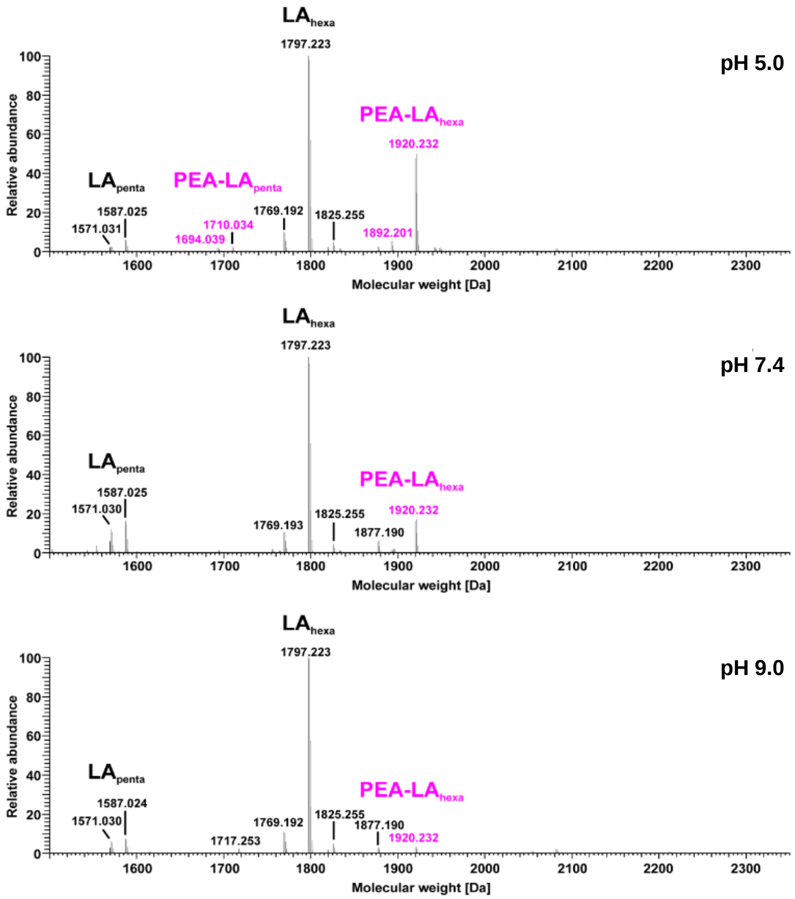

FIG S5 Enzymatic activities of MCR-1 are pH-dependent. Charge-deconvoluted MS spectra of lipid A derived from *in vitro* reconstitution assays of PEA-modification of lipid A using purified MCR-1, PE and LPS under different pH conditions. The shown *m/z*-region comprises penta- to hepta-acylated lipid A species. MCR-1 PEA-transfer activity is clearly observed at pH 5 and pH 7.4, with a significant increase at acidified pH. Unmodified lipid A species are labelled in black, PEA-modified species in pink. Spectra shown are representative of three independent biological replicates.

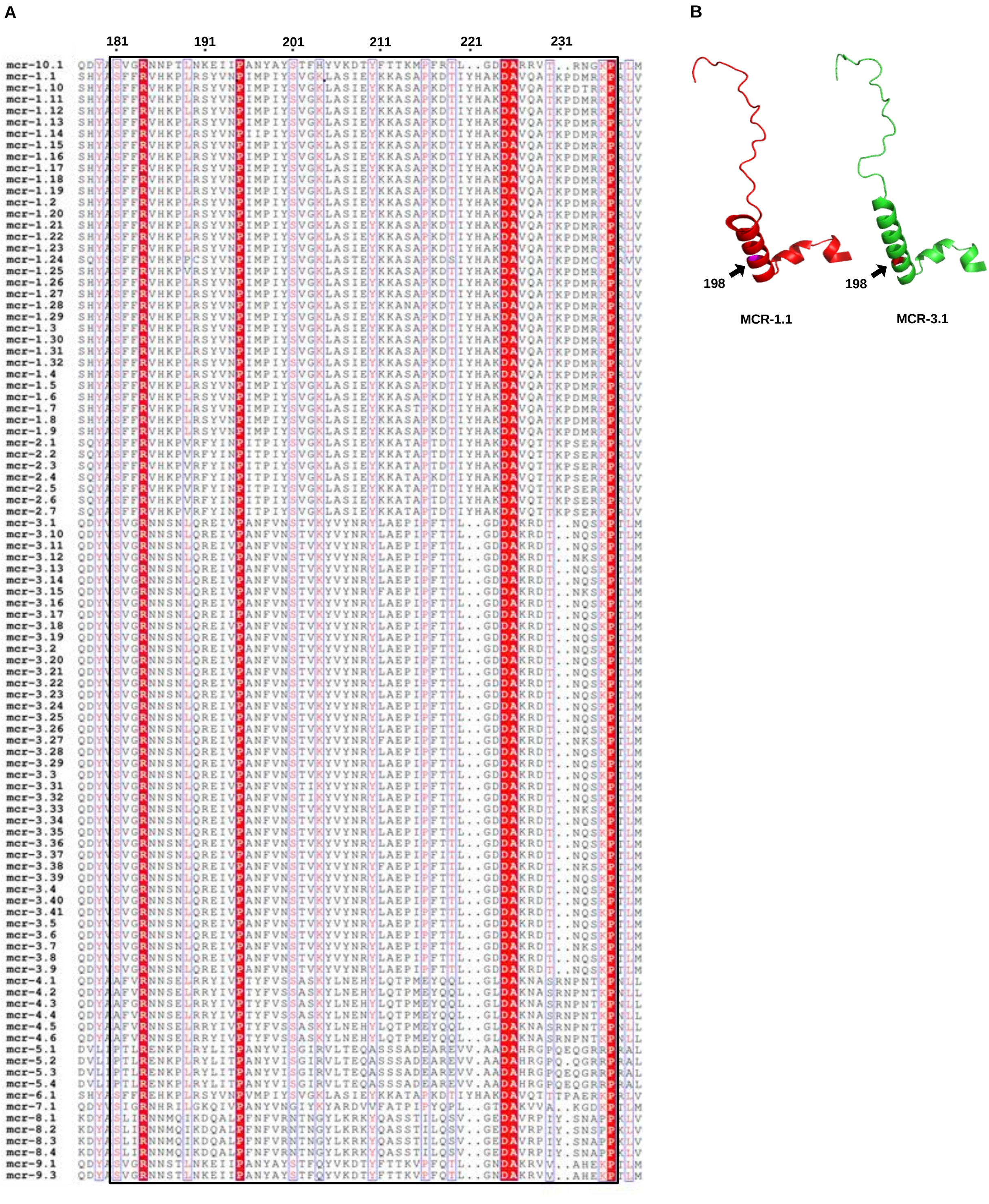

FIG S6 (A) Multiple sequence alignments of the PBD of different *mcr*-alleles. Comparison of amino acids at region 181-237 of different *mcr*-alleles. (B) Comparative structure analysis of the PBD of MCR-1 and MCR-3.

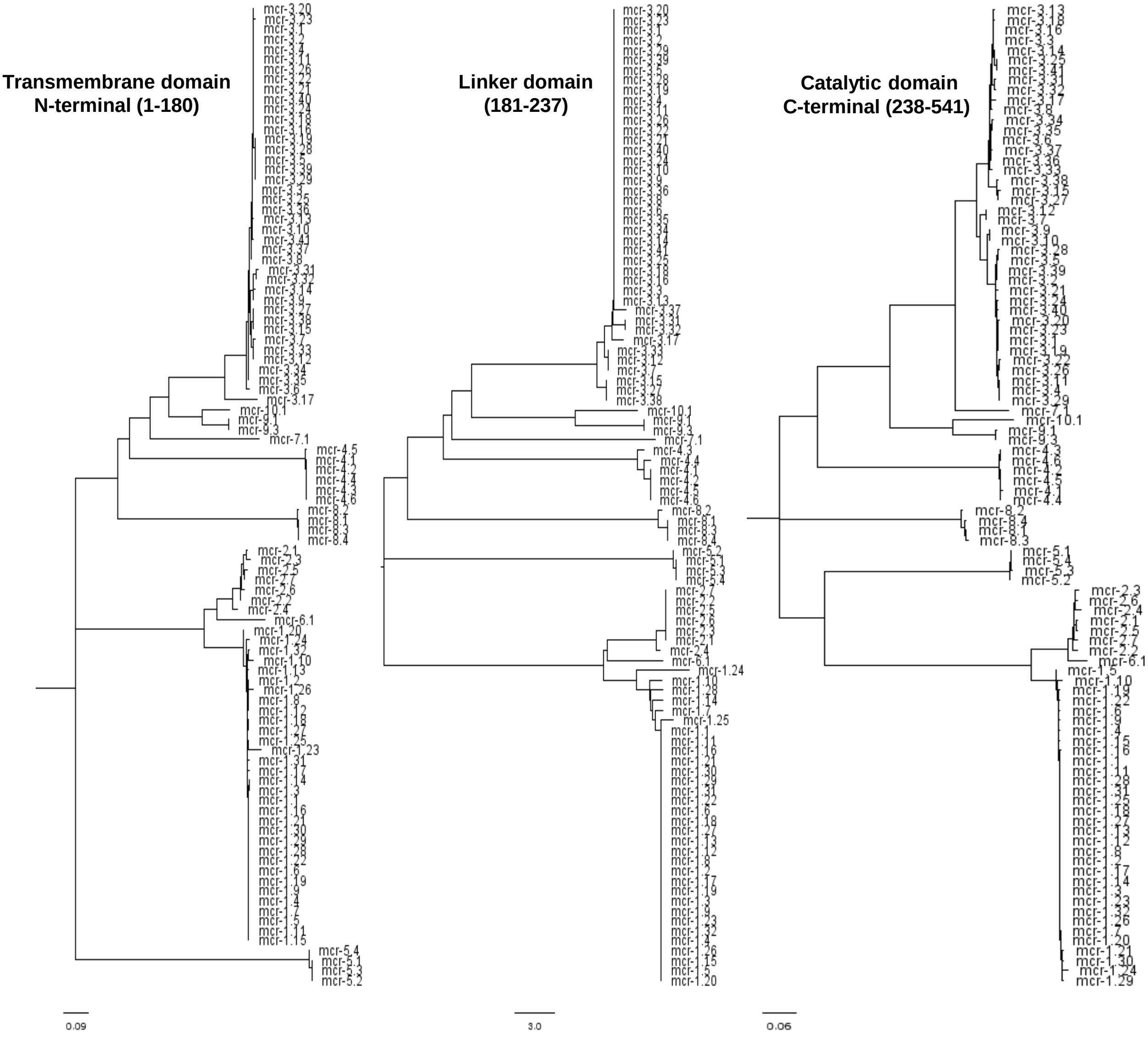

FIG S7 Phylogenetic tree of MCR-family. Comparison of *mcr*-allele based on the number of differences in amino acids in the transmembrane, linker and catalytic domain. Irrespective of domain, each *mcr*-allele clustered together indicating their integrity.

Proteinsequenzanalyse HZI-SFPR

Probennummer: B4314 Auftraggeber: Hudel Datum:21.03.2018

Abteilung: SBA VAX: MedMik Gie

Probenbezeichnung: Probe 1

Probenzustand: geblottet auf: PVDF

Probenmenge: Probenvolumen:

Sequencer: ABI 494A Procise HT

Cartridge: Blot C

Methode: PL PVDF Protein

Sequenz:

1	2	3	4	5	6	7	8	9	10	
**I**	**Y**	**S**	**V**	**G**	**K**	**L**	**A**	**S**	**I**	
9.4	8.7	6.4	6.4	4.4	1.5	5.1	3.6	1.7	2.8	pmol
L	A	?	I	E	Y	?	?	?	?	
4.3	4.8		2.0	1.8	1.9					pmol
		E				A				

11	12	13	14	15	16	17	18	19	20

Identifizierung: Pos.1 in der Sequenz ab Pos. 198

Bemerkung:

Cycle 2:Standard 1

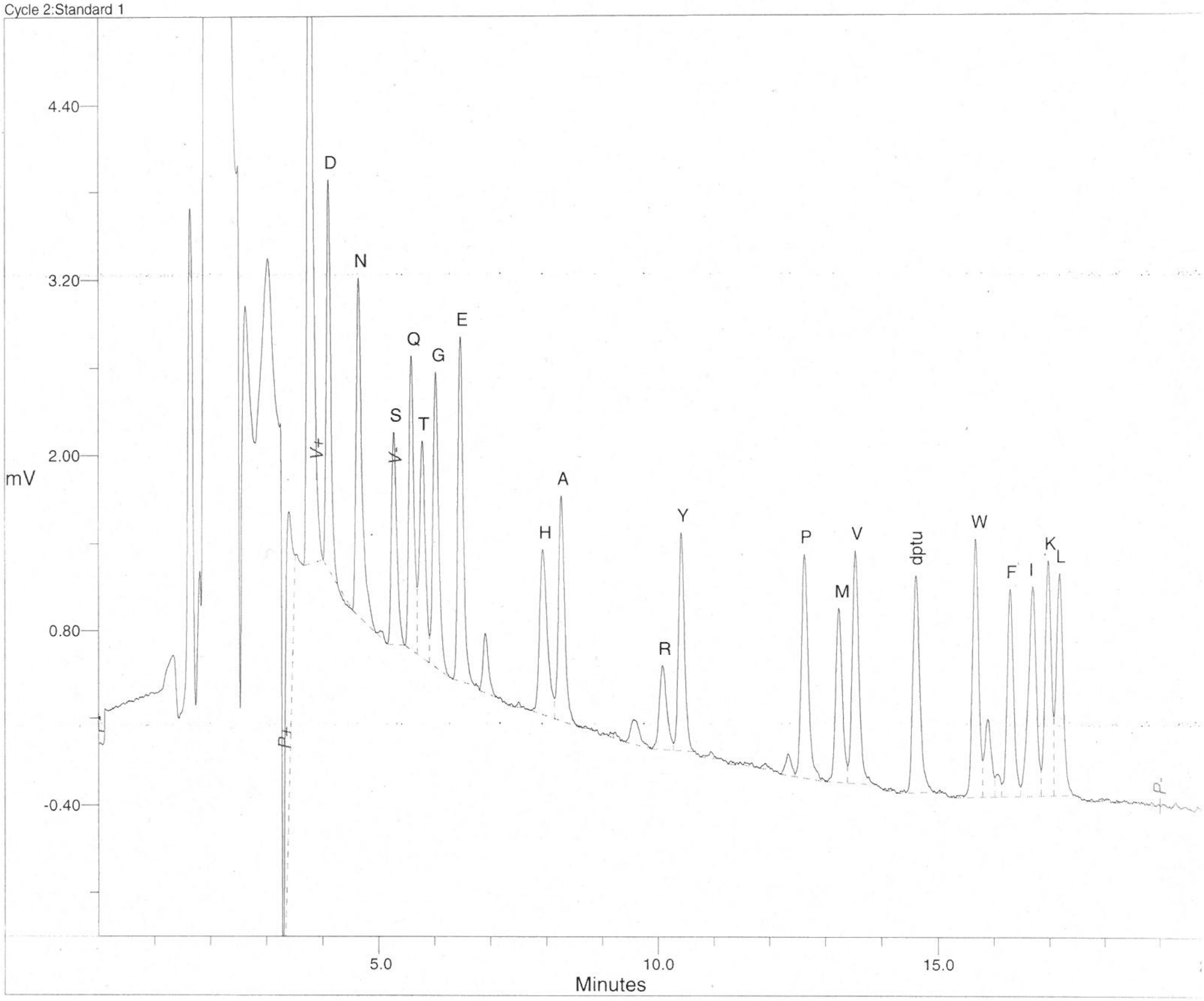

PEAK ID	R.TIME (mins)	C.TIME (mins)	HEIGHT (mV)	PMOL HT
	3.40		1.992	
	3.77		6.372	
D	4.10	4.10	2.660	2.000
N	4.64	4.64	2.311	2.000
	5.03		0.041	
S	5.27	5.27	1.454	2.000
Q	5.58	5.58	2.018	2.000
T	5.78	5.78	1.484	2.000
G	6.01	6.01	2.023	2.000
E	6.46	6.46	2.364	2.000
	6.90		0.411	
	7.38		0.019	
	7.50		0.036	
H	7.93	7.93	1.136	2.000
A	8.26	8.26	1.546	2.000
	8.70		0.016	
	8.80		0.017	
	9.01		0.015	
	9.13		0.031	
	9.22		0.045	
	9.55		0.170	
R	10.07	10.07	0.577	2.000
Y	10.41	10.41	1.498	2.000
	10.80		0.015	
	10.94		0.047	
	11.30		0.024	
	11.40		0.014	

PEAK ID	R.TIME (mins)	C.TIME (mins)	HEIGHT (mV)	PMOL HT
	11.55		0.023	
	11.68		0.030	
	11.93		0.039	
	12.33		0.149	
P	12.62	12.62	1.538	2.000
M	13.24	13.24	1.196	2.000
V	13.53	13.53	1.598	2.000
	14.06		0.016	
	14.16		0.022	
	14.40		0.022	
dptu	14.61	14.61	1.489	2.000
	15.03		0.020	
	15.27		0.015	
	15.39		0.019	
W	15.65	15.65	1.774	2.000
	15.89		0.533	
	16.06		0.159	
F	16.28	16.28	1.425	2.000
I	16.69	16.69	1.439	2.000
K	16.97	16.97	1.616	2.000
L	17.17	17.17	1.526	2.000
	17.76		0.018	
	17.94		0.020	
	18.02		0.011	
	18.23		0.020	
	18.35		0.021	
	18.85		0.024	

Cycle 3:Residue 1

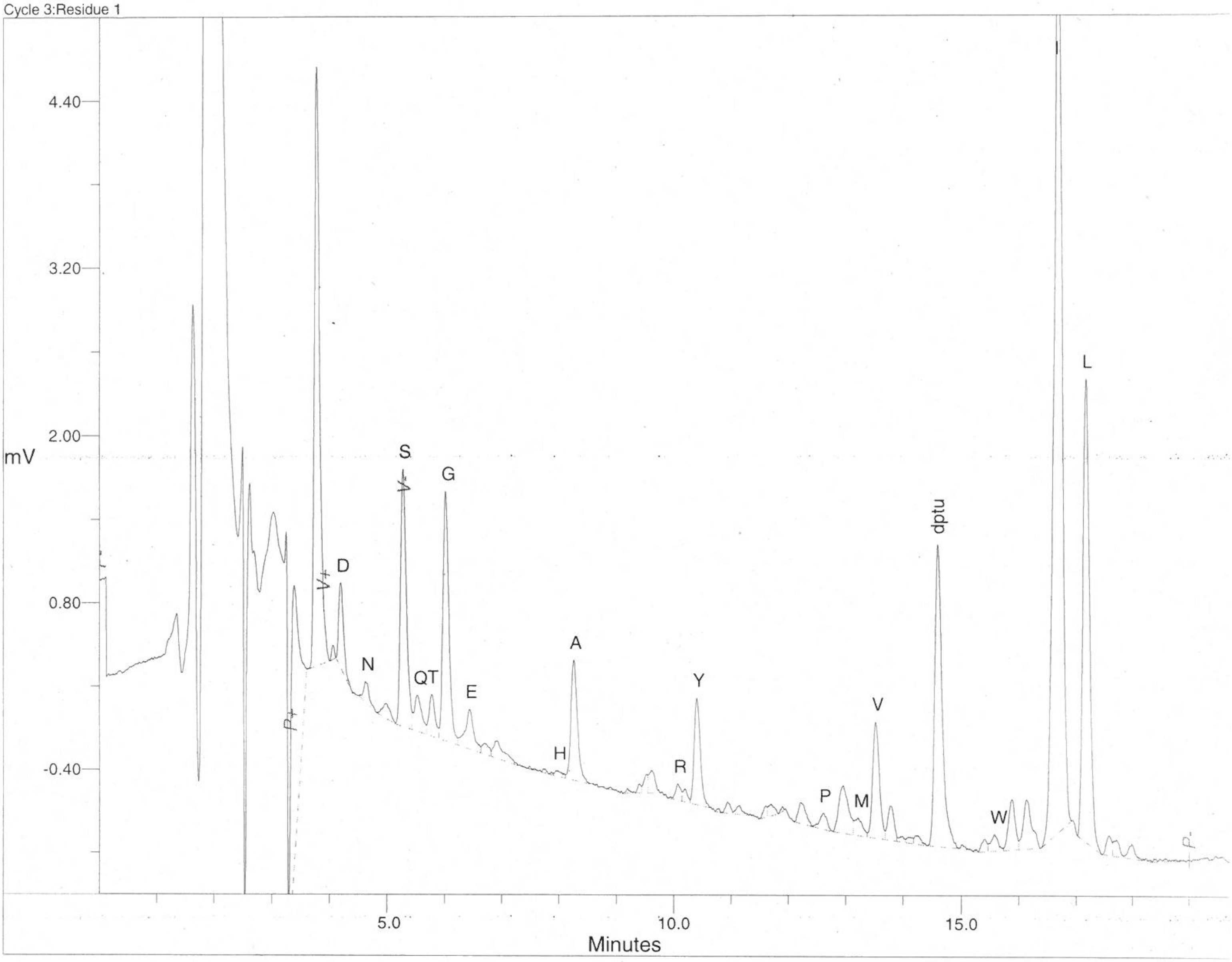

PEAK ID	R.TIME (mins)	C.TIME (mins)	HEIGHT (mV)	PMOL HT
	3.39		2.058	
	3.77		4.304	
	4.06		0.103	
D	4.19	4.10	0.611	0.459
N	4.63	4.64	0.147	0.127
	4.99		0.108	
S	5.28	5.27	1.846	2.539
Q	5.53	5.58	0.236	0.234
T	5.78	5.78	0.299	0.403
G	6.02	6.01	1.794	1.773
E	6.44	6.46	0.286	0.242
	6.71		0.082	
	6.92		0.127	
	7.48		0.023	
	7.57		0.013	
	7.72		0.028	
	7.87		0.021	
H	7.97	7.93	0.041	0.072
A	8.25	8.26	0.861	1.114
	8.88		0.019	
	9.19		0.034	
	9.39		0.067	
	9.52		0.136	
	9.60		0.163	
R	10.07	10.07	0.120	0.418
	10.19		0.096	
Y	10.40	10.41	0.769	1.026
	10.78		0.031	
	10.93		0.082	
	11.14		0.066	
	11.60		0.081	

PEAK ID	R.TIME (mins)	C.TIME (mins)	HEIGHT (mV)	PMOL HT
	11.70		0.084	
	11.89		0.037	
	12.21		0.152	
P	12.61	12.62	0.117	0.153
	12.95		0.346	
M	13.22	13.24	0.127	0.213
V	13.52	13.53	0.839	1.050
	13.78		0.249	
	13.97		0.037	
	14.12		0.047	
	14.23		0.064	
dptu	14.60	14.61	2.171	2.916
	15.00		0.030	
	15.23		0.018	
	15.40		0.092	
W	15.56	15.65	0.123	0.139
	15.87		0.366	
	16.13		0.358	
I	16.68	16.69	6.763	9.401
L	17.16	17.17	3.314	4.342
	17.57		0.144	
	17.71		0.124	
	17.99		0.109	
	18.19		0.012	
	18.33		0.015	
	18.38		0.025	
	18.49		0.021	
	18.60		0.021	
	18.71		0.011	
	18.76		0.016	
	18.85		0.010	

Cycle 4:Residue 2

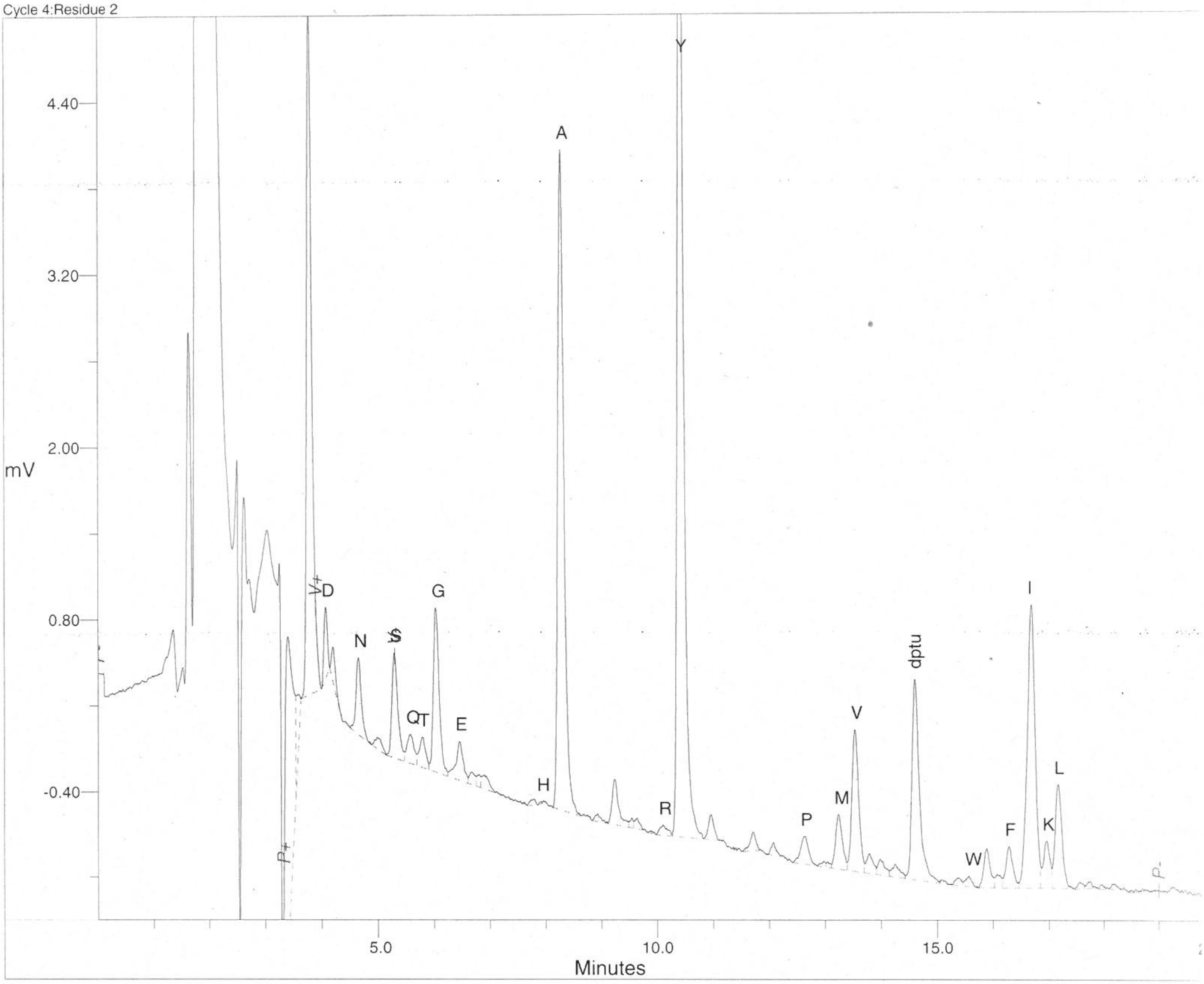

PEAK ID	R.TIME (mins)	C.TIME (mins)	HEIGHT (mV)	PMOL HT
	3.40		2.215	
	3.60		0.285	
	3.78		4.771	
D	4.07	4.10	0.493	0.371
	4.20		0.240	
N	4.65	4.64	0.532	0.460
	4.98		0.079	
S	5.29	5.27	0.730	1.004
Q	5.58	5.58	0.202	0.201
T	5.79	5.78	0.212	0.286
G	6.03	6.01	1.142	1.129
E	6.45	6.46	0.273	0.231
	6.67		0.088	
	6.80		0.087	
	6.90		0.101	
	7.54		0.012	
	7.79		0.043	
H	7.91	7.93	0.015	0.027
A	8.27	8.26	4.589	5.938
	8.93		0.055	
	9.23		0.319	
	9.54		0.069	
	9.63		0.074	
	9.88		0.018	
R	10.10	10.07	0.074	0.256
Y	10.41	10.41	7.349	9.811
	10.95		0.177	
	11.36		0.018	

PEAK ID	R.TIME (mins)	C.TIME (mins)	HEIGHT (mV)	PMOL HT
	11.71		0.131	
	12.07		0.093	
P	12.63	12.62	0.198	0.258
	12.97		0.041	
	13.03		0.039	
M	13.24	13.24	0.386	0.645
V	13.53	13.53	0.995	1.245
	13.78		0.124	
	13.98		0.111	
	14.24		0.089	
dptu	14.60	14.61	1.404	1.885
	15.07		0.029	
	15.38		0.054	
W	15.57	15.65	0.075	0.085
	15.88		0.277	
	16.07		0.098	
F	16.28	16.28	0.292	0.410
I	16.68	16.69	1.975	2.746
K	16.95	16.97	0.309	0.382
L	17.16	17.17	0.730	0.956
	17.55		0.048	
	17.73		0.049	
	17.95		0.033	
	18.16		0.040	
	18.49		0.015	
	18.59		0.021	
	18.70		0.021	
	18.91		0.008	

Cycle 5:Residue 3

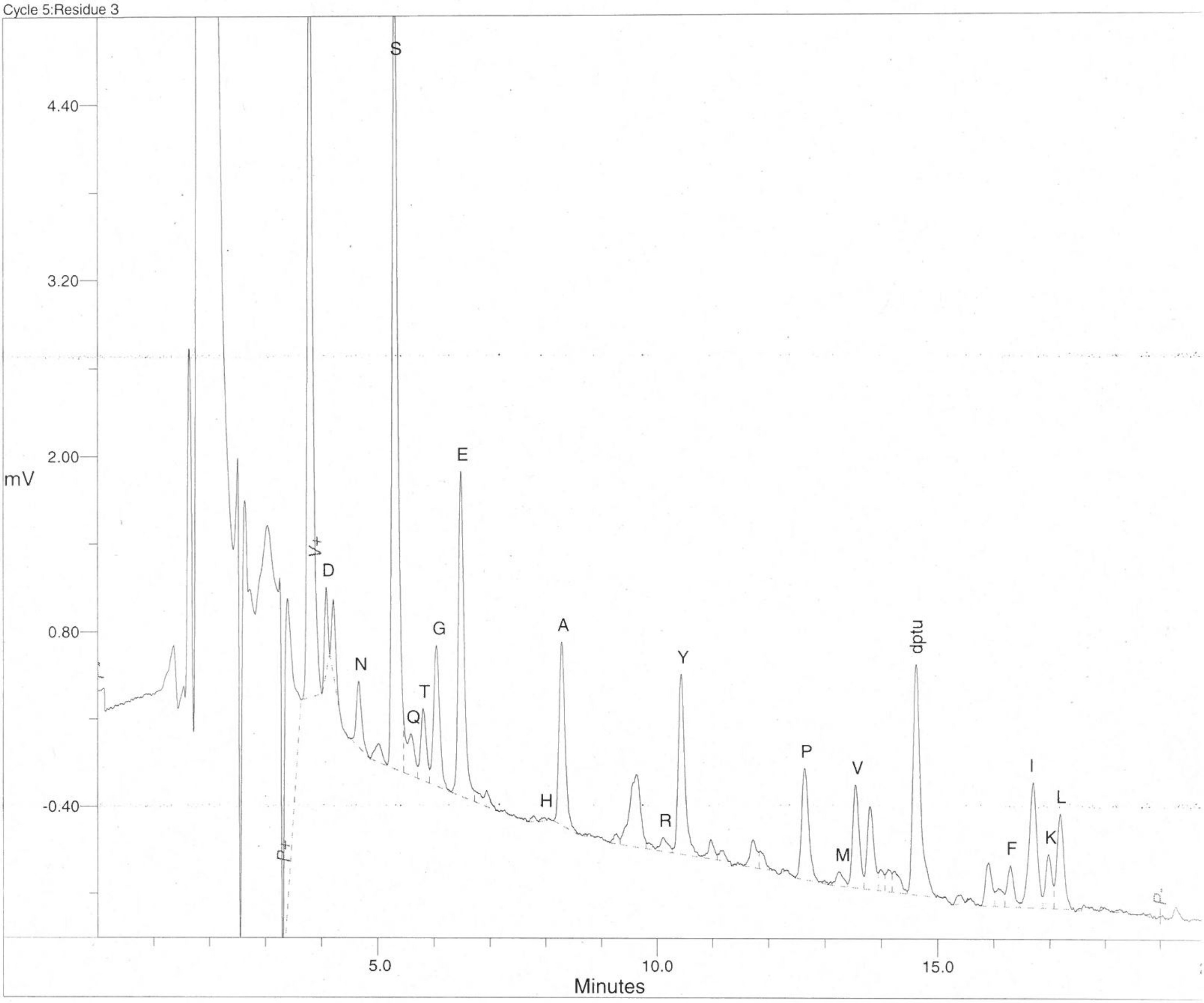

PEAK ID	R.TIME (mins)	C.TIME (mins)	HEIGHT (mV)	PMOL HT
	3.40		2.124	
	3.80		5.600	
D	4.09	4.10	0.581	0.437
	4.22		0.515	
N	4.66	4.64	0.457	0.395
	5.03		0.131	
S	5.30	5.27	5.447	7.490
Q	5.58	5.58	0.247	0.245
T	5.80	5.78	0.497	0.670
G	6.04	6.01	0.969	0.958
E	6.48	6.46	2.227	1.884
	6.95		0.113	
	7.64		0.017	
	7.79		0.044	
H	7.97	7.93	0.022	0.039
A	8.28	8.26	1.262	1.633
	8.71		0.017	
	8.79		0.020	
	9.27		0.073	
	9.63		0.495	
	9.85		0.028	
R	10.10	10.07	0.096	0.333
Y	10.43	10.41	1.238	1.652
	10.96		0.134	
	11.18		0.072	
	11.45		0.019	
	11.72		0.190	

PEAK ID	R.TIME (mins)	C.TIME (mins)	HEIGHT (mV)	PMOL HT
	11.85		0.121	
	12.25		0.024	
P	12.64	12.62	0.762	0.991
	12.99		0.024	
	13.10		0.023	
M	13.26	13.24	0.098	0.165
V	13.55	13.53	0.705	0.882
	13.80		0.567	
	14.00		0.148	
	14.13		0.155	
	14.24		0.148	
dptu	14.62	14.61	1.583	2.127
	15.40		0.062	
	15.53		0.017	
	15.91		0.298	
	16.08		0.126	
F	16.30	16.28	0.286	0.402
I	16.70	16.69	0.856	1.190
K	16.98	16.97	0.372	0.461
L	17.18	17.17	0.651	0.852
	17.54		0.017	
	17.60		0.034	
	17.93		0.024	
	18.12		0.013	
	18.27		0.024	
	18.81		0.025	
	18.89		0.018	

Cycle 6:Residue 4

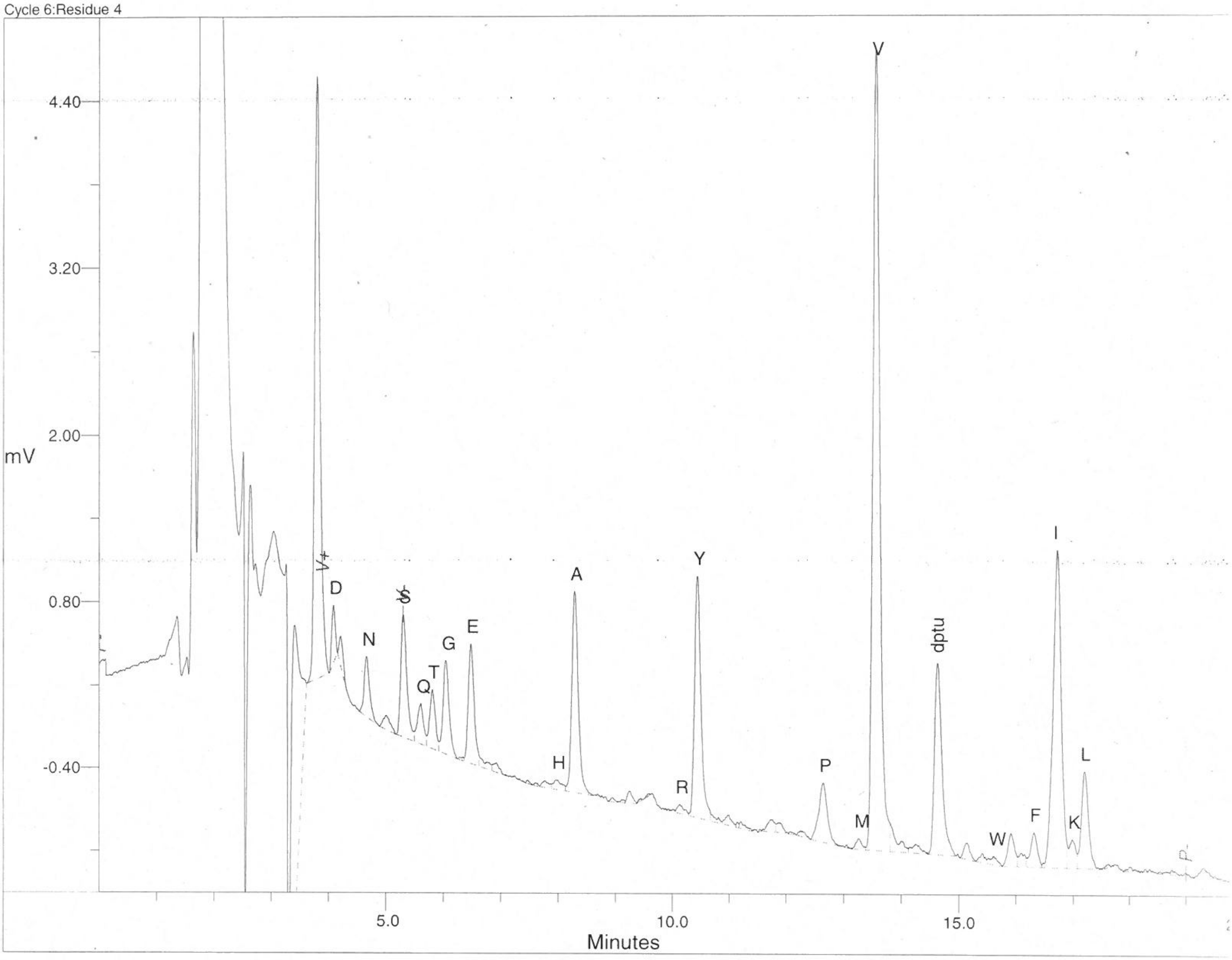

PEAK ID	R.TIME (mins)	C.TIME (mins)	HEIGHT (mV)	PMOL HT
	3.41		2.226	
	3.79		4.333	
D	4.09	4.10	0.412	0.310
	4.21		0.207	
N	4.66	4.64	0.435	0.377
	5.01		0.104	
S	5.30	5.27	0.888	1.221
Q	5.60	5.58	0.294	0.292
T	5.81	5.78	0.430	0.580
G	6.04	6.01	0.681	0.673
	6.29		0.020	
E	6.48	6.46	0.868	0.734
	6.76		0.045	
	6.92		0.068	
	7.45		0.022	
	7.60		0.022	
	7.77		0.051	
H	7.97	7.93	0.070	0.123
A	8.29	8.26	1.451	1.878
	8.95		0.030	
	9.11		0.022	
	9.25		0.089	
	9.45		0.018	
	9.58		0.018	
	9.88		0.013	
	10.00		0.018	
R	10.13	10.07	0.067	0.234
Y	10.44	10.41	1.743	2.327
	10.81		0.039	
	10.99		0.077	
	11.14		0.038	

PEAK ID	R.TIME (mins)	C.TIME (mins)	HEIGHT (mV)	PMOL HT
	11.20		0.050	
	11.48		0.022	
	11.55		0.016	
	11.76		0.091	
	11.86		0.071	
	12.07		0.018	
	12.25		0.044	
P	12.65	12.62	0.428	0.557
	13.05		0.023	
M	13.26	13.24	0.078	0.131
V	13.56	13.53	5.757	7.205
	14.00		0.069	
	14.24		0.067	
dptu	14.63	14.61	1.382	1.857
	15.14		0.118	
	15.40		0.067	
W	15.60	15.65	0.059	0.066
	15.91		0.239	
	16.08		0.098	
F	16.31	16.28	0.249	0.349
I	16.71	16.69	2.283	3.173
K	16.98	16.97	0.172	0.213
L	17.20	17.17	0.696	0.912
	17.61		0.026	
	18.02		0.030	
	18.29		0.023	
	18.37		0.019	
	18.54		0.015	
	18.64		0.018	
	18.75		0.039	

Cycle 7:Residue 5

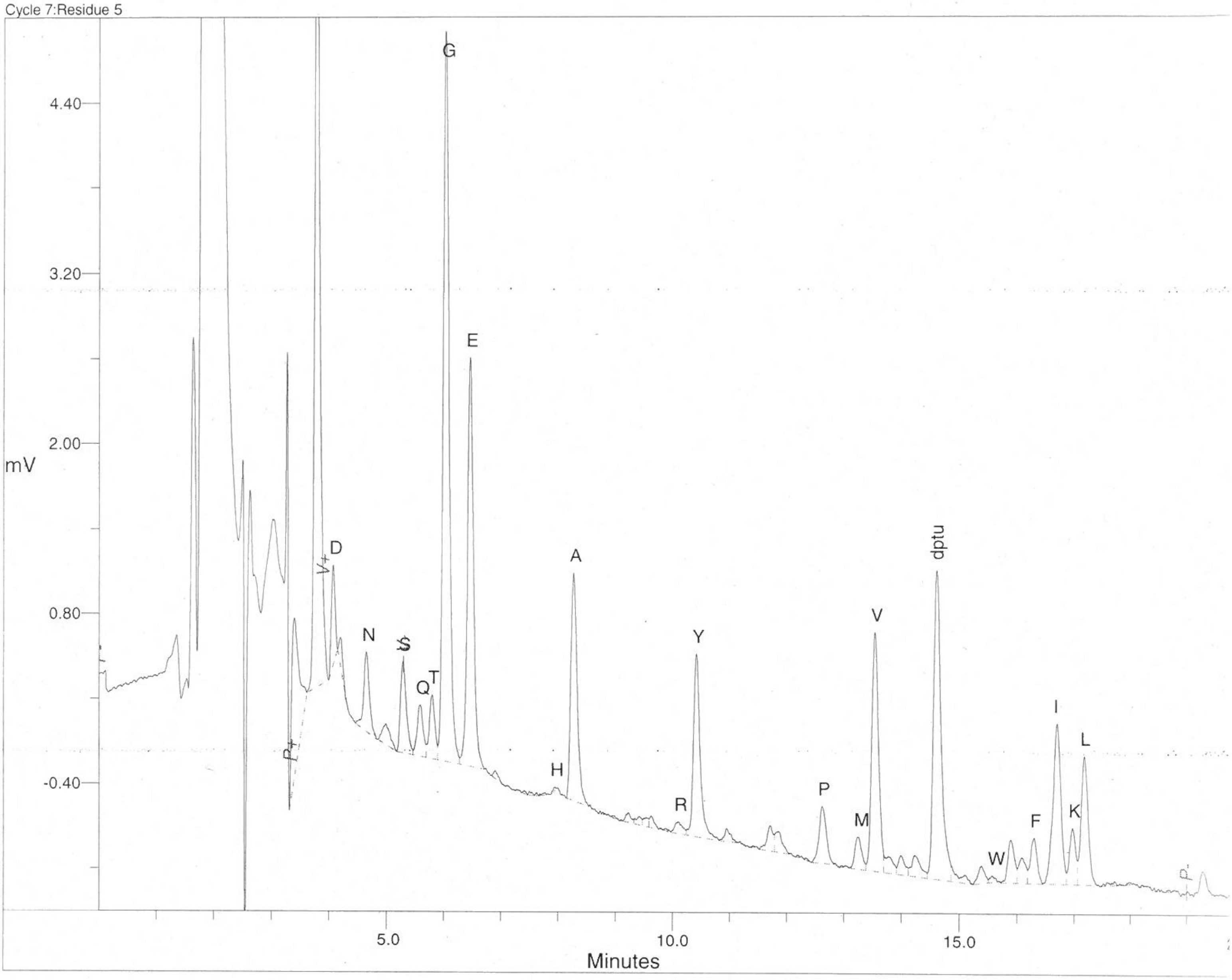

PEAK ID	R.TIME (mins)	C.TIME (mins)	HEIGHT (mV)	PMOL HT
	3.40		1.128	
	3.78		5.759	
D	4.08	4.10	0.710	0.534
	4.20		0.190	
N	4.65	4.64	0.561	0.485
	4.99		0.147	
S	5.29	5.27	0.642	0.882
Q	5.58	5.58	0.361	0.358
T	5.80	5.78	0.448	0.604
G	6.03	6.01	5.143	5.083
E	6.46	6.46	2.886	2.442
	6.90		0.055	
	7.40		0.023	
	7.50		0.027	
	7.63		0.022	
	7.78		0.017	
H	7.94	7.93	0.028	0.050
A	8.27	8.26	1.609	2.082
	8.73		0.020	
	8.97		0.017	
	9.10		0.009	
	9.22		0.069	
	9.41		0.061	
	9.53		0.063	
	9.63		0.082	
	9.87		0.016	
R	10.10	10.07	0.083	0.289
Y	10.41	10.41	1.287	1.718
	10.94		0.092	
	11.40		0.011	

PEAK ID	R.TIME (mins)	C.TIME (mins)	HEIGHT (mV)	PMOL HT
	11.71		0.179	
	11.86		0.147	
	12.18		0.013	
	12.33		0.016	
P	12.62	12.62	0.403	0.523
	12.97		0.014	
M	13.25	13.24	0.229	0.382
V	13.54	13.53	1.683	2.107
	13.78		0.091	
	13.99		0.137	
	14.24		0.151	
dptu	14.61	14.61	2.180	2.928
	15.09		0.054	
	15.38		0.126	
W	15.56	15.65	0.049	0.056
	15.89		0.308	
	16.08		0.185	
F	16.30	16.28	0.323	0.453
I	16.70	16.69	1.132	1.573
K	16.97	16.97	0.400	0.495
L	17.18	17.17	0.906	1.187
	17.52		0.021	
	17.59		0.026	
	17.72		0.030	
	17.98		0.018	
	18.22		0.015	
	18.50		0.021	
	18.68		0.017	
	18.95		0.011	

Cycle 8:Residue 6

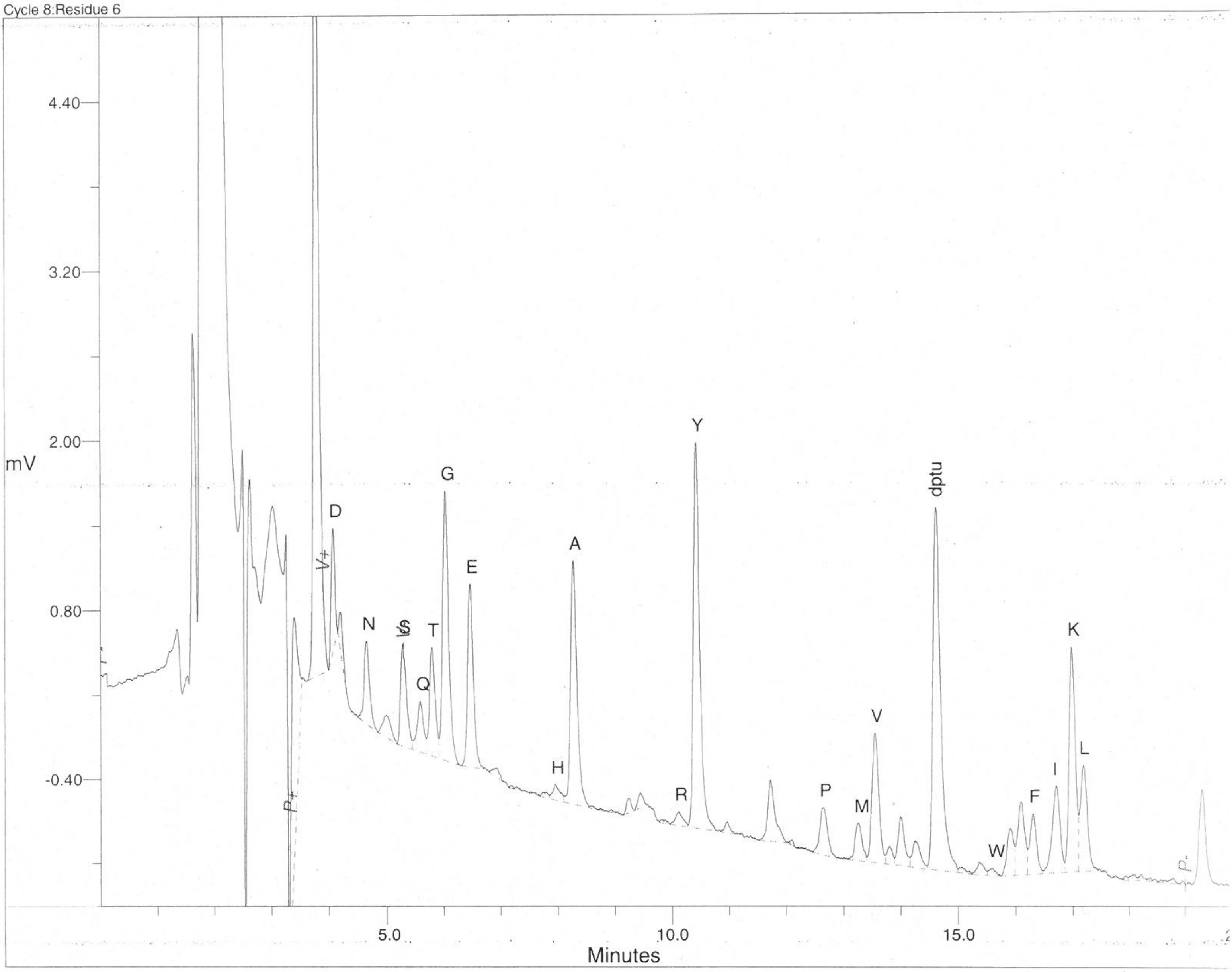

PEAK ID	R.TIME (mins)	C.TIME (mins)	HEIGHT (mV)	PMOL HT
	3.40		1.674	
	3.78		6.021	
D	4.07	4.10	0.872	0.656
	4.20		0.271	
N	4.65	4.64	0.584	0.506
	4.99		0.158	
S	5.29	5.27	0.721	0.992
Q	5.58	5.58	0.361	0.358
T	5.79	5.78	0.767	1.034
G	6.03	6.01	1.910	1.888
E	6.46	6.46	1.296	1.097
	6.91		0.061	
	7.18		0.021	
	7.28		0.024	
	7.52		0.013	
	7.79		0.041	
H	7.95	7.93	0.111	0.195
A	8.27	8.26	1.730	2.238
	8.58		0.017	
	8.74		0.014	
	8.93		0.020	
	9.25		0.101	
	9.44		0.105	
	9.81		0.026	
	9.88		0.027	
R	10.11	10.07	0.101	0.350
Y	10.42	10.41	2.730	3.645
	10.81		0.009	
	10.96		0.078	
	11.71		0.442	

PEAK ID	R.TIME (mins)	C.TIME (mins)	HEIGHT (mV)	PMOL HT
	12.08		0.035	
	12.21		0.011	
P	12.64	12.62	0.339	0.440
	13.00		0.013	
M	13.25	13.24	0.257	0.429
V	13.55	13.53	0.894	1.119
	13.80		0.084	
	13.99		0.306	
	14.25		0.160	
dptu	14.62	14.61	2.558	3.436
	15.05		0.034	
	15.20		0.021	
	15.36		0.092	
W	15.58	15.65	0.054	0.061
	15.90		0.339	
	16.08		0.525	
F	16.30	16.28	0.435	0.611
I	16.70	16.69	0.623	0.865
K	16.98	16.97	1.596	1.976
L	17.18	17.17	0.760	0.996
	17.86		0.013	
	17.93		0.034	
	18.10		0.046	
	18.22		0.046	
	18.38		0.026	
	18.57		0.022	
	18.63		0.023	
	18.80		0.039	
	18.95		0.023	

Cycle 9:Residue 7

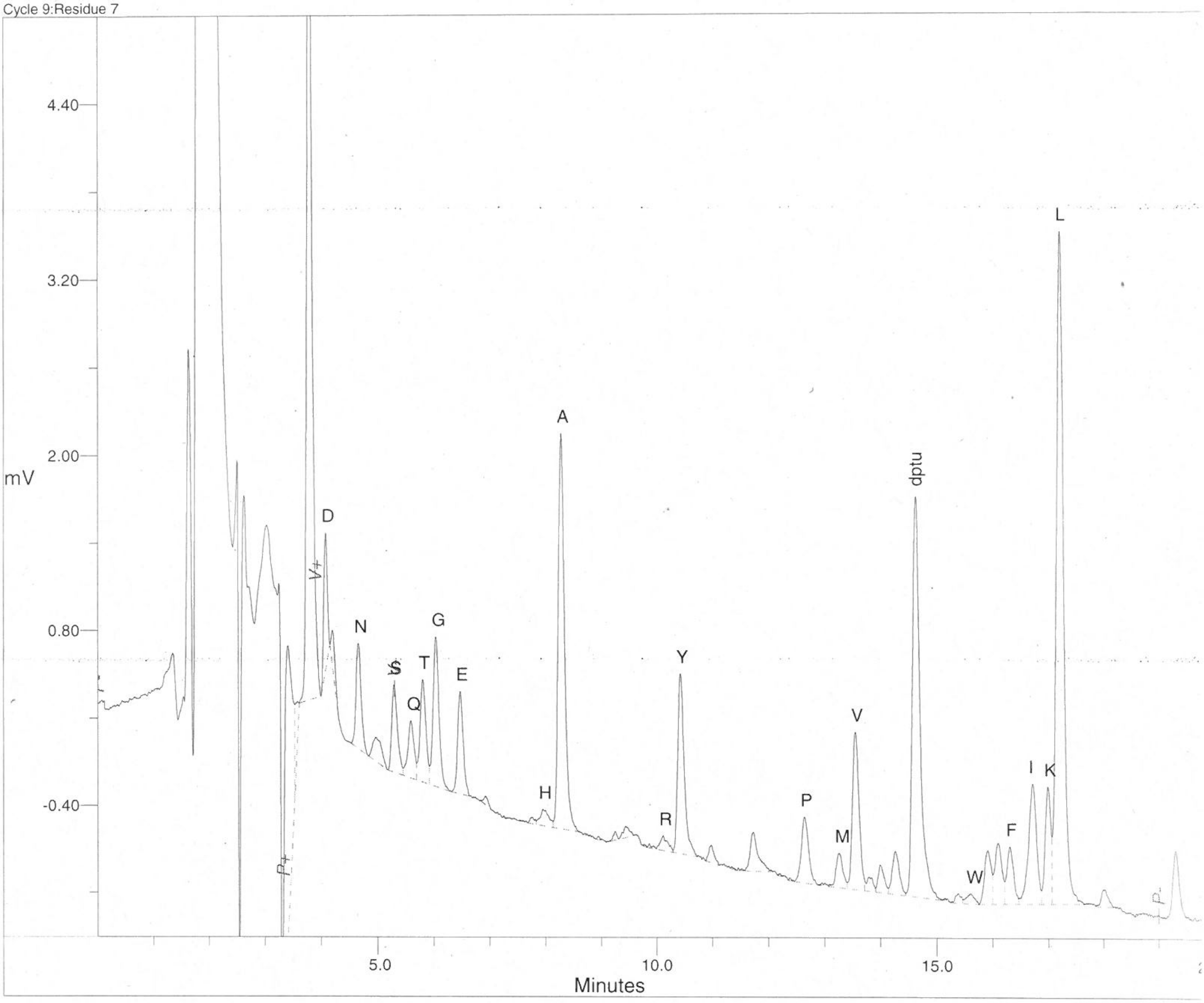

PEAK ID	R.TIME (mins)	C.TIME (mins)	HEIGHT (mV)	PMOL HT
	3.40		2.061	
	3.58		0.260	
	3.78		6.064	
D	4.08	4.10	0.942	0.708
	4.20		0.230	
N	4.65	4.64	0.719	0.622
	4.96		0.165	
S	5.29	5.27	0.597	0.822
Q	5.59	5.58	0.392	0.389
T	5.80	5.78	0.704	0.948
G	6.03	6.01	1.027	1.015
E	6.47	6.46	0.705	0.596
	6.92		0.061	
	7.25		0.016	
	7.54		0.008	
	7.76		0.047	
H	7.95	7.93	0.113	0.200
A	8.28	8.26	2.710	3.507
	8.69		0.011	
	8.94		0.022	
	9.25		0.060	
	9.43		0.079	
	9.77		0.012	
	9.86		0.030	
	9.94		0.019	
R	10.11	10.07	0.102	0.354
Y	10.42	10.41	1.237	1.652

PEAK ID	R.TIME (mins)	C.TIME (mins)	HEIGHT (mV)	PMOL HT
	10.97		0.120	
	11.34		0.015	
	11.46		0.020	
	11.52		0.015	
	11.72		0.267	
	12.19		0.015	
	12.38		0.020	
P	12.64	12.62	0.454	0.591
	12.94		0.015	
M	13.25	13.24	0.237	0.396
V	13.55	13.53	1.083	1.356
	13.79		0.080	
	13.99		0.192	
	14.26		0.295	
dptu	14.62	14.61	2.742	3.682
	15.13		0.012	
	15.38		0.067	
W	15.60	15.65	0.069	0.078
	15.90		0.367	
	16.09		0.418	
F	16.30	16.28	0.390	0.548
I	16.70	16.69	0.827	1.149
K	16.99	16.97	0.806	0.998
L	17.19	17.17	4.589	6.013
	17.68		0.009	
	18.01		0.118	
	18.60		0.015	

Cycle 10:Residue 8

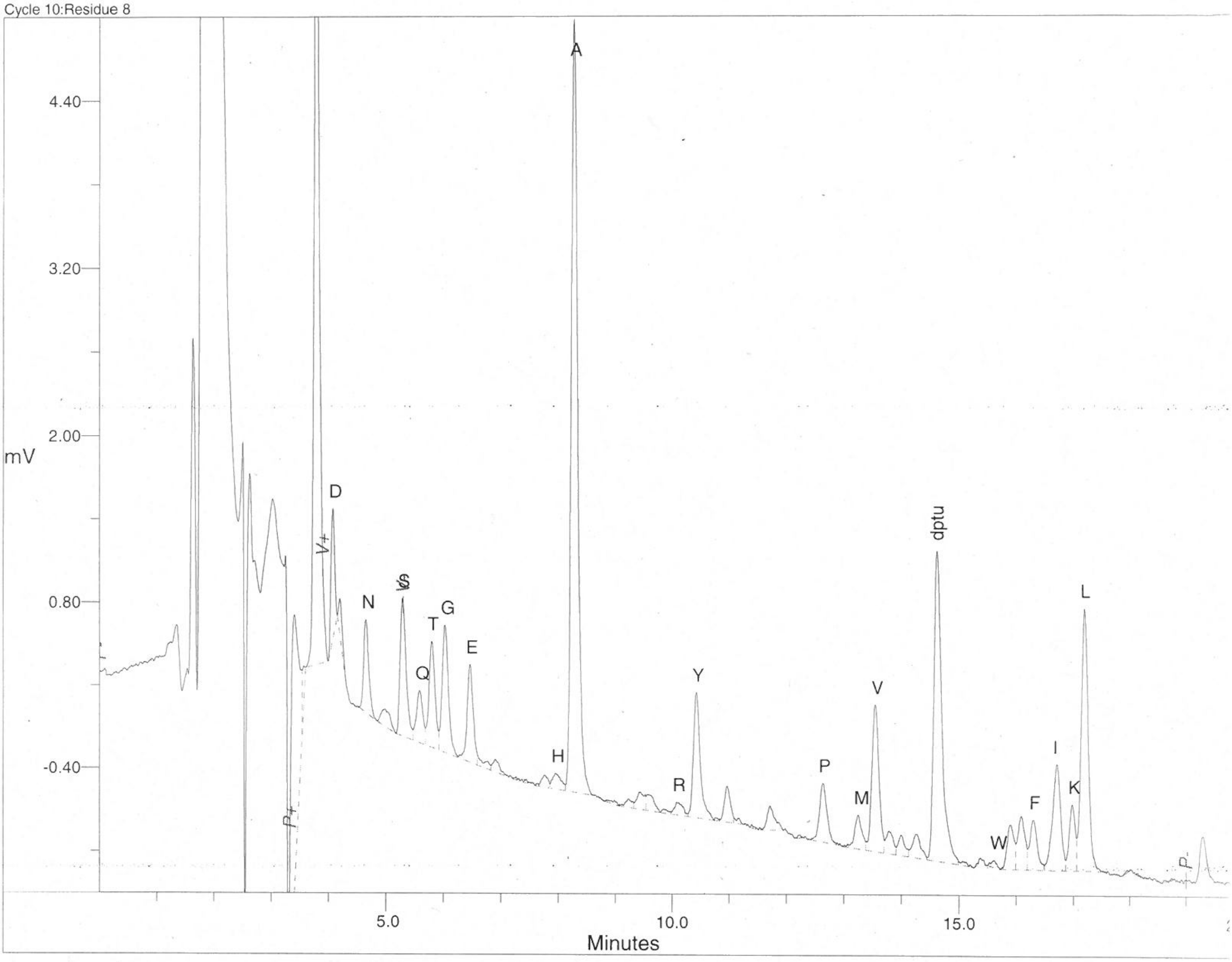

PEAK ID	R.TIME (mins)	C.TIME (mins)	HEIGHT (mV)	PMOL HT
	3.40		2.053	
	3.57		0.283	
	3.78		6.112	
D	4.08	4.10	0.933	0.702
	4.20		0.269	
	4.52		0.010	
N	4.65	4.64	0.674	0.584
	4.97		0.122	
S	5.28	5.27	0.994	1.367
Q	5.58	5.58	0.377	0.374
T	5.79	5.78	0.768	1.035
G	6.03	6.01	0.922	0.911
E	6.46	6.46	0.709	0.600
	6.76		0.051	
	6.90		0.091	
	7.26		0.022	
	7.45		0.027	
	7.64		0.018	
	7.77		0.088	
H	7.95	7.93	0.114	0.200
A	8.27	8.26	5.549	7.180
	8.85		0.015	
	8.99		0.030	
	9.07		0.015	
	9.23		0.063	
	9.44		0.126	
	9.56		0.116	
	9.85		0.031	
R	10.08	10.07	0.091	0.317
Y	10.42	10.41	0.905	1.208
	10.76		0.021	

PEAK ID	R.TIME (mins)	C.TIME (mins)	HEIGHT (mV)	PMOL HT
	10.97		0.259	
	11.16		0.032	
	11.39		0.026	
	11.54		0.017	
	11.70		0.174	
	12.23		0.024	
P	12.63	12.62	0.424	0.551
	13.01		0.015	
	13.07		0.025	
M	13.25	13.24	0.245	0.409
V	13.55	13.53	1.060	1.327
	13.78		0.139	
	14.00		0.146	
	14.25		0.170	
dptu	14.62	14.61	2.231	2.996
	15.05		0.021	
	15.37		0.031	
W	15.61	15.65	0.053	0.060
	15.90		0.319	
	16.08		0.384	
F	16.29	16.28	0.357	0.501
I	16.70	16.69	0.762	1.059
K	16.97	16.97	0.473	0.586
L	17.18	17.17	1.890	2.477
	17.95		0.018	
	18.14		0.018	
	18.26		0.009	
	18.64		0.017	
	18.75		0.017	
	18.91		0.020	

Cycle 11:Residue 9

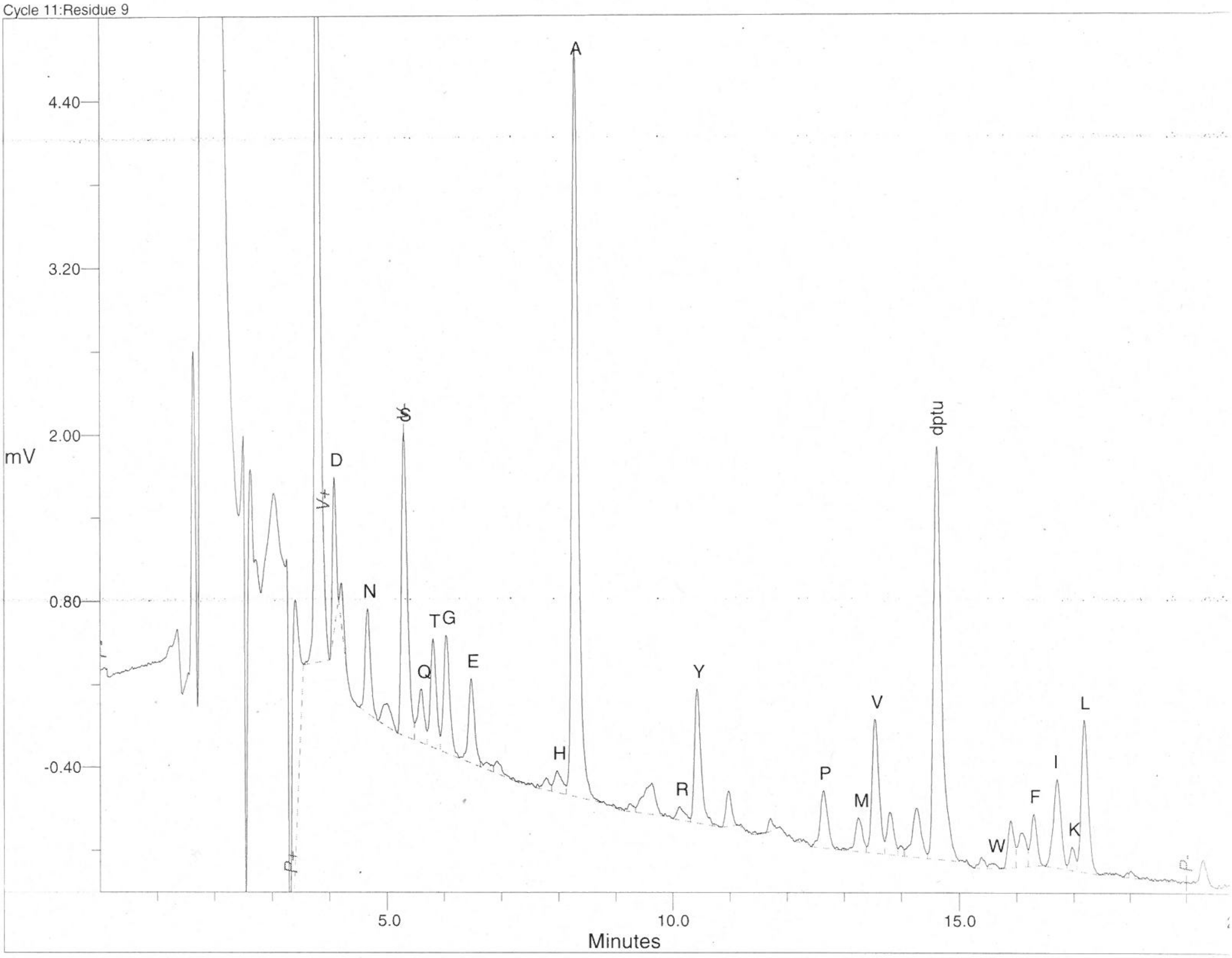

PEAK ID	R.TIME (mins)	C.TIME (mins)	HEIGHT (mV)	PMOL HT
	3.41		1.842	
	3.79		6.435	
D	4.09	4.10	1.092	0.821
	4.20		0.274	
N	4.66	4.64	0.754	0.652
	5.00		0.168	
S	5.30	5.27	2.189	3.010
Q	5.60	5.58	0.393	0.389
T	5.81	5.78	0.792	1.067
G	6.04	6.01	0.855	0.845
E	6.47	6.46	0.616	0.521
	6.73		0.045	
	6.92		0.091	
	7.20		0.018	
	7.30		0.013	
	7.47		0.012	
	7.63		0.029	
	7.78		0.089	
H	7.97	7.93	0.158	0.279
A	8.28	8.26	5.385	6.968
	8.95		0.019	
	9.05		0.013	
	9.25		0.058	
	9.63		0.228	
	9.85		0.018	
R	10.12	10.07	0.097	0.336
Y	10.43	10.41	0.969	1.293
	10.97		0.254	
	11.18		0.018	
	11.43		0.022	
	11.54		0.008	

PEAK ID	R.TIME (mins)	C.TIME (mins)	HEIGHT (mV)	PMOL HT
	11.70		0.091	
	12.23		0.025	
	12.29		0.024	
P	12.64	12.62	0.410	0.534
	12.86		0.017	
	12.97		0.017	
M	13.25	13.24	0.241	0.404
V	13.55	13.53	0.963	1.206
	13.80		0.304	
	13.98		0.075	
	14.28		0.352	
dptu	14.62	14.61	2.969	3.988
	15.13		0.029	
	15.38		0.082	
W	15.57	15.65	0.039	0.044
	15.90		0.338	
	16.08		0.247	
F	16.30	16.28	0.372	0.523
I	16.70	16.69	0.636	0.884
K	16.97	16.97	0.171	0.211
L	17.18	17.17	1.093	1.433
	17.58		0.017	
	17.64		0.016	
	17.95		0.036	
	18.02		0.050	
	18.21		0.012	
	18.38		0.015	
	18.45		0.017	
	18.62		0.020	
	18.75		0.016	
	18.95		0.017	

Cycle 12:Residue 10

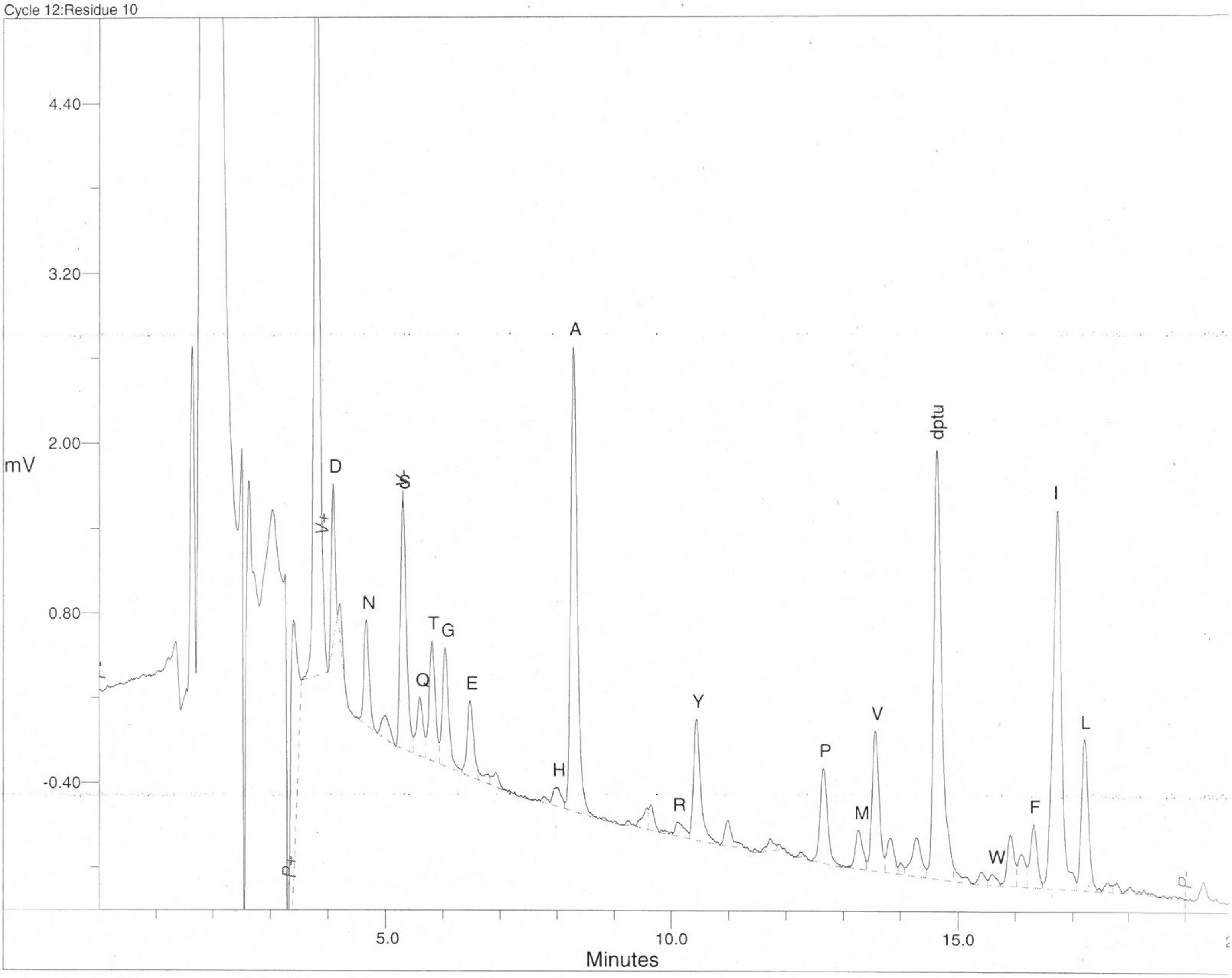

PEAK ID	R.TIME (mins)	C.TIME (mins)	HEIGHT (mV)	PMOL HT
	3.40		1.833	
	3.79		6.373	
D	4.09	4.10	1.132	0.851
	4.20		0.209	
	4.50		0.012	
N	4.66	4.64	0.743	0.643
	4.98		0.162	
S	5.30	5.27	1.768	2.431
Q	5.60	5.58	0.415	0.411
T	5.81	5.78	0.851	1.147
G	6.04	6.01	0.838	0.828
E	6.47	6.46	0.537	0.454
	6.79		0.067	
	6.92		0.103	
	7.26		0.017	
	7.43		0.015	
	7.76		0.043	
H	7.99	7.93	0.136	0.240
A	8.28	8.26	3.287	4.253
	8.81		0.017	
	9.01		0.016	
	9.25		0.033	
	9.57		0.155	
	9.64		0.179	
	9.82		0.024	
	9.98		0.021	
R	10.10	10.07	0.101	0.349
Y	10.43	10.41	0.857	1.144
	10.98		0.178	
	11.45		0.025	

PEAK ID	R.TIME (mins)	C.TIME (mins)	HEIGHT (mV)	PMOL HT
	11.72		0.090	
	11.87		0.042	
	12.25		0.036	
P	12.65	12.62	0.672	0.874
M	13.27	13.24	0.275	0.459
V	13.56	13.53	0.990	1.239
	13.82		0.248	
	14.00		0.087	
	14.28		0.276	
dptu	14.63	14.61	3.023	4.060
	15.12		0.039	
	15.42		0.088	
W	15.60	15.65	0.074	0.084
	15.91		0.351	
	16.10		0.213	
F	16.32	16.28	0.421	0.591
I	16.73	16.69	2.649	3.682
L	17.20	17.17	1.054	1.381
	17.48		0.019	
	17.62		0.067	
	17.78		0.070	
	18.02		0.037	
	18.19		0.022	
	18.27		0.035	
	18.36		0.022	
	18.61		0.017	
	18.73		0.018	
	18.81		0.023	
	18.91		0.021	

Table S3

Antibodies	Description	Source
Monoclonal mouse antibody C12	Anti-MCR-1 antibody	This work
Monoclonal mouse antibody F6	Anti-MCR-1 antibody	This work
Monoclonal mouse antibody A5	Anti-MCR-1 antibody	This work
Monoclonal mouse antibody C4	Anti-MCR-1 antibody	This work
m-IgGkBP-HRP	Mouse IgGκ light chain binding protein conjugated to HRP	Santa Cruz Biotechnology, Inc., Dallas, United States

Antibiotics	Source
Colistin sulfate	Bela-Pharm GmbH & Co.KG, Vechta
Ampicillin salt	Carl Roth GmbH & Co. KG, Karlsruhe
Chloramphenicol	Sigma Aldrich Chemie GmbH, Steinheim
Kanamycin sulfate	Sigma Aldrich Chemie GmbH, Steinheim
Tetracycline hydrochloride	Sigma Aldrich Chemie GmbH, Steinheim

Bacterial strains	Description	Antibiotic resistance	Source
E. coli MG1655	*E. coli* K12 strain		Lab stock
E. coli DH5α	*E. coli* K12 strain		Lab stock
E. coli DH10β	*E. coli* K12 strain		Lab stock
E. coli BL21 (DE3)	*E. coli* B strain for protein expression (T7 promoter-based system)		Lab stock
E. coli DH10β pSIM5-tet	*E. coli* strain used for gene deletion	Tetracycline	B. Berghoff (Gift)
E. coli DH10β pKD4	*E. coli* strain harboring the template for gene deletion	Amp/Kan	Addgene
E. coli DH10β pCP20	*E. coli* strain harboring the yeast Flp recombinase gene	Amp/Cm	Addgene
E. coli DH10β pUC19	*E. coli* strain harboring pUC19	Ampicillin	This work
E. coli DH10β pUC19::*mcr-1*	*E. coli* strain expressing *mcr-1*	Ampicillin	This work
E. coli DH10β pUC19::*mcr-1*$_{P198A}$	*E. coli* strain expressing *mcr-1*$_{P198A}$	Ampicillin	This work
E. coli DH10β pUC19::*mcr-1*$_{P198Y}$	*E. coli* strain expressing *mcr-1*$_{P198Y}$	Ampicillin	This work
E. coli DH10β pUC19::*mcr-1*$_{H478A}$	*E. coli* strain expressing *mcr-1*$_{H478A}$	Ampicillin	This work
E. coli DH10β Δ*degP*	*degP* deletion mutant		This work
E. coli DH10β Δ*degP* pUC19	*degP* deletion mutant harboring pUC19	Ampicillin	This work
E. coli DH10β Δ*degP* pUC19::*mcr-1*	*degP* deletion mutant expressing *mcr-1*	Ampicillin	This work
E. coli DH10β Δ*degP* pUC19::*mcr-1*$_{P198A}$	*degP* deletion mutant expressing *mcr-1*$_{P198A}$	Ampicillin	This work
E. coli DH10β Δ*degP* pUC19::*mcr-1*$_{P198Y}$	*degP* deletion mutant expressing *mcr-1*$_{P198Y}$	Ampicillin	This work
E. coli DH10β Δ*degP* pUC19::*mcr-1*$_{H478A}$	*degP* deletion mutant expressing *mcr-1*$_{H478A}$	Ampicillin	This work
E. coli DH10β Δ*cpxRA*	Δ*cpxRA* deletion mutant		This work
E. coli DH10β Δ*cpxRA* pUC19	Δ*cpxRA* deletion mutant harboring pUC19	Ampicillin	This work
E. coli DH10β Δ*cpxRA* pUC19::*mcr-1*	Δ*cpxRA* deletion mutant expressing *mcr-1*	Ampicillin	This work
E. coli DH10β Δ*cpxRA* pUC19::*mcr-1*$_{P198A}$	Δ*cpxRA* deletion mutant expressing *mcr-1*$_{P198A}$	Ampicillin	This work
E. coli DH10β Δ*cpxRA* pUC19::*mcr-1*$_{P198Y}$	Δ*cpxRA* deletion mutant expressing *mcr-1*$_{P198Y}$	Ampicillin	This work
E. coli DH10β Δ*cpxRA* pUC19::*mcr-1*$_{H478A}$	Δ*cpxRA* deletion mutant expressing *mcr-1*$_{H478A}$	Ampicillin	This work
E. coli DH10β Δ*tolC*	*tolC* deletion mutant		This work
E. coli DH10β Δ*tolC* pUC19	*tolC* deletion mutant harboring pUC19	Ampicillin	This work
E. coli DH10β Δ*tolC* pUC19::*mcr-1*	*tolC* deletion mutant expressing *mcr-1*	Ampicillin	This work
E. coli DH10β Δ*tolC* pV163M	*tolC* deletion mutant expressing *mcr-1* encoded on pV163M	Kanamycin	This work
E. coli DH10β Δ*tolC* pV163M::Δ*mcr-1*	*tolC* deletion mutant harboring pV163M lacking *mcr-1*	Kanamycin	This work
E. coli BL21 (DE3) pET-28 a (+)	*E. coli* B strain harboring pET-28 a (+)	Kanamycin	This work
E. coli BL21 (DE3) pET-28 a (+)::*mcr-1*	*E. coli* B strain expressing *mcr-1*	Kanamycin	This work
E. coli BL21 (DE3) pET-28 a (+)::*mcr-1*$_{P198A}$	*E. coli* B strain expressing *mcr-1*$_{P198A}$	Kanamycin	This work
E. coli BL21 (DE3) pET-28 a (+)::*mcr-1*$_{H478A}$	*E. coli* B strain expressing *mcr-1*$_{H478A}$	Kanamycin	This work
E. coli PAD282	β-Gal reporter strain		T. Silhavy (Gift)
E. coli PAD282 pUC19	β-Gal reporter strain harboring pUC19	Ampicillin	This work
E. coli PAD282 pUC19::*mcr-1*	β-Gal reporter strain expressing *mcr-1*	Ampicillin	This work
E. coli PAD282 pUC19::*mcr-1*$_{P198A}$	β-Gal reporter strain expressing *mcr-1*$_{P198A}$	Ampicillin	This work
E. coli PAD282 pUC19::*mcr-1*$_{P198Y}$	β-Gal reporter strain expressing *mcr-1*$_{P198Y}$	Ampicillin	This work
E. coli PAD282 pUC19::*mcr-1*$_{H478A}$	β-Gal reporter strain expressing *mcr-1*$_{H478A}$	Ampicillin	This work
E. coli PAD282 pUC19::*mcr-1* + pSU2719::*degP*	β-Gal reporter strain expressing *mcr-1* and exogenous DegP	Amp/Cm	This work
E. coli PAD282 pUC19::*mcr-1*$_{H478A}$ + pSU2719::*degP*	β-Gal reporter strain expressing *mcr-1*$_{H478A}$ and exogenous DegP	Amp/Cm	This work
E. coli DH10β Δ*degP* pUC19::*mcr-1* + pSU2719::*degP*	Complementation of *degP* deletion mutant expressing *mcr-1*	Amp/Cm	This work
E. coli DH10β Δ*degP* pUC19::*mcr-1*$_{H478A}$ + pSU2719::*degP*	Complementation of *degP* deletion mutant expressing *mcr-1*$_{H478A}$	Amp/Cm	This work

Bacterial strains	Description	Antibiotic resistance	Source
V163	MCR-1 producing clinical *E. coli*-isolate. Source of pV163M	Colistin resistant	Falgenhauer et al. 2016
NRZ14408	MCR-1 producing clinical *E. coli*-isolate	Colistin resistant	Falgenhauer et al. 2016
E. coli 023	MCR-1 producing clinical *E. coli*-isolate	Colistin resistant	Imirzalioglu et al. 2017
E. coli 053	MCR-1 producing clinical *E. coli*-isolate	Colistin resistant	Imirzalioglu et al. 2017
E. coli 032	MCR-1 producing clinical *E. coli*-isolate	Colistin resistant	Imirzalioglu et al. 2017
SurvCare230	Non-MCR-1 producing clinical *E. coli*-isolate	Colistin susceptible	SurvCare study
SurvCare221	Non-MCR-1 producing clinical *E. coli*-isolate	Colistin susceptible	SurvCare study
SurvCare254	Non-MCR-1 producing clinical *E. coli*-isolate	Colistin susceptible	SurvCare study
**Commercial kits**	**Source**		
Q5 Site-Directed Mutagenesis Kit	New England Biolabs GmbH, Frankfurt am Main		
Gibson Assembly Cloning Kit	New England Biolabs GmbH, Frankfurt am Main		
PureLink Genomic DNA Mini Kit	Thermo Fisher Scientific, Langenselbold		
MSB Spin PCRapace	STRATEC Molecular GmbH, Berlin		
GenElute Plasmid Miniprep Kit	Sigma Aldrich Chemie GmbH, Steinheim		
PureLink Quick Gel Extraction Kit	Thermo Fisher Scientific, Langenselbold		
BCA Protein Assay Kit	Thermo Fisher Scientific, Langenselbold		
Bradford Protein Assay Kit	Bio-Rad Laboratories, Inc., California, United States		
miRNeasy Mini Kit	QIAGEN GmbH, Hilden		
Quanti Tect SYBR Green PCR Kit	QIAGEN GmbH, Hilden		
**Critical chemicals, reagents and peptides**	**Source**		
Bile salts	Sigma Aldrich Chemie GmbH, Steinheim		
IPTG	Carl Roth GmbH & Co. KG, Karlsruhe		
Bacto Yeast Extract	Becton Dickinson GmbH, Heidelberg		
Bacto Tryptone	Becton Dickinson GmbH, Heidelberg		
Bacto Agar	Becton Dickinson GmbH, Heidelberg		
Mueller Hinton II Broth (Cation-Ajusted)	Sigma Aldrich Chemie GmbH, Steinheim		
*Sal*I	New England Biolabs GmbH, Frankfurt am Main		
*Eco*RI	New England Biolabs GmbH, Frankfurt am Main		
Q5 High-Fidelity DNA Polymerase	New England Biolabs GmbH, Frankfurt am Main		
Taq DNA Polymerase	Thermo Fisher Scientific, Langenselbold		
1 Kb Plus DNA ladder	Thermo Fisher Scientific, Langenselbold		
dNTPs	Thermo Fisher Scientific, Langenselbold		
Endoproteinase Glu-C from *S. aureus* V8	Sigma Aldrich Chemie GmbH, Steinheim		
Acrylamide/Bis-acrylamide, 30% solution	Carl Roth GmbH + Co. KG, Karlsruhe		
Coomassie brilliant blue R-250	Sigma Aldrich Chemie GmbH, Steinheim		
Skim Milk Powder	Sigma Aldrich Chemie GmbH, Steinheim		
Protein-Marker V ('Prestained'), peqGOLD	Avantor, Inc., Radnor, United States		
Prestained Protein Ladder	Abcam, Cambridge, Great Britain		
PageRuler Plus Prestained Protein Ladder	Thermo Fisher Scientific, Langenselbold		
BlueEasy Prestained Protein Marker	NIPPON Genetics EUROPE, Düren		
PVDF Western Blotting Membranes	Roche Diagnostics GmbH, Mannheim		
Imidazole	Merck KGaA, Darmstadt		
ECL Western Blotting Substrate	Promega GmbH, Walldorf		
Peptide DYFGSALLRV	Intavis Peptide Services GmbH & Co. KG, Tübingen		
Antimicrobial peptide LL-37	GL Biochem (Shanghai) Ltd., China		
Phosphate-buffered saline (PBS)	Sigma Aldrich Chemie GmbH, Steinheim		
o-nitrophenyl-β-D-galactopyranoside (ONPG)	Thermo Fisher Scientific, Langenselbold		
Acyl 12:0 NBD-PE	Avanti Polar Lipids, Inc., Alabaster, United States		
TLC Silica gel 60	Merck KGaA, Darmstadt		
L-α-Phosphatidylethanolamine from *Glycine max* (soybean)	Avanti Polar Lipids, Inc., Alabaster, United States		
Dodecyl-β-D-maltosid (DDM)	Carl Roth GmbH + Co. KG, Karlsruhe		
Isopropyl-β-D-thiogalactopyranosid (IPTG)	Carl Roth GmbH + Co. KG, Karlsruhe		
Amido Black	SERVA Electrophoresis GmbH, Heidelberg		
Protease inhibitor mix	SERVA Electrophoresis GmbH, Heidelberg		
RNAprotect	QIAGEN GmbH, Hilden		
Super Script II Reverse Transcriptase	Thermo Fisher Scientific, Langenselbold		

Oligonucleotides	Sequence 5`→3`
*mcr-1*_pUC19_F	GCGCGCGTCGACATGATGCAGCATACTTCTGTGT
*mcr-1*_pUC19_R	GCGCGCGAATTCTCAGCGGATGAATGCGGTGC
*mcr-1*_pET-28 a (+)_F	CTTTAAGAAGGAGATATACCATGATGCAGCATACTTCTGTGTG
*mcr-1*_pET-28 a (+)_R	CAGTGGTGGTGGTGGTGGTGGCGGATGAATGCGGTGCG
pET-28 a (+)_F	CACCACCACCACCACCACTG
pET-28 a (+)_R	GGTATATCTCCTTCTTAAAGTTAAACAAAATTATTTCTAGAGGGGAATTGTTATC
*mcr-1*_Mut_P198A_F	TCCGATCATGGCAATCTACTC
*mcr-1*_Mut_P198A_R	TTGACATAGCTACGCAGC
*mcr-1*_Mut_P198Y_F	TCCGATCATGTACATCTACTCGGTGG
*mcr-1*_Mut_P198Y_R	TTGACATAGCTACGCAGC
*mcr-1*_Mut_H478A_F	TGTCTATCTAGCAGGTATGCCAAATGCCTTTGC
*mcr-1*_Mut_H478A_R	CCGTTCTCACCCAGACTT
*degP*_pSU2719_F	GTACCCGGGGATCCTCTAGATTACTGCATTAACAGGTAG
*degP*_pSU2719_R	GCTAATGTGGTTTTTTTCATCAAGCTTGGCGTAATCATGG
pSU2719_F	CAAGCTTGGCGTAATCATGG
pSU2719_R	GTACCCGGGGATCCTCTAGA
Del_*cpxRA*_F	CGTCTGATGACGTAATTTCTGCCTCGGAGGTATTTAAACAGTGTAGGCTGGAGCTGCTTC
Del_*cpxRA*_R	ATGCGGCGTAAACGCCTTATCCTGCCTGCAAATGCGAAGTCATATGAATATCCTCCTTAG
control_*cpxRA*_F	CAGCAGCGTGGCTTAATGA
control_*cpxRA*_R	GATGACGATCAATATCACCG
Del_*degP*_F	ACAGCAATTTTGCGTTATCTGTTAATCGAGACTGAAATACGTGTAGGCTGGAGCTGCTTC
Del_*degP*_R	GGAGAACCCCTTCCCGTTTTCAGGAAGGGGTTGAGGGAGACATATGAATATCCTCCTTAG
control_*degP*_F	ATCAGCGGTATGACCGACC
control_*degP*_R	GCCACGATATCCTGCGCCAT
Del_*tolC*_F	TTTGATCGCGCTAAATACTGCTTCACCACAAGGAATGCAAGTGTAGGCTGGAGCTGCTTC
Del_*tolC*_R	GCCTTACGTTCAGACGGGGCCGAAGCCCCGTCGTCGTCATCATATGAATATCCTCCTTAG
control_*tolC*_F	TGCCAAATGTAACGGGCAGG
control_*tolC*_R	CAGCTGAACAGTCGTCAGCA
qRT-PCR_MCR-1_F	TCATGCCAATCTACTCGGTG
qRT-PCR_MCR-1_R	ATTGGTCACGCCATCGATC
qRT-PCR-16S-rRNA_F	ACAGGATTAGATACCCTGGT
qRT-PCR-16S-rRNA_R	ACTTCCGTGGATGTCAAGAC

Plasmids	Description	Antibiotic resistance	Source
pUC19	Cloning vector	Ampicillin	Lab stock
pUC19::*mcr-1*	pUC19 encoding *mcr-1*	Ampicillin	This work
pUC19::*mcr-1*$_{P198A}$	pUC19 encoding *mcr-1*$_{P198A}$	Ampicillin	This work
pUC19::*mcr-1*$_{P198Y}$	pUC19 encoding *mcr-1*$_{P198Y}$	Ampicillin	This work
pUC19::*mcr-1*$_{H478A}$	pUC19 encoding *mcr-1*$_{H478A}$	Ampicillin	This work
pSU2719	Cloning vector	Chloramphenicol	Lab stock
pSU2719::*degP*	pSU2719 encoding *degP*	Chloramphenicol	This work
pET-28 a (+)	Expression vector	Kanamycin	Lab stock
pET-28 a (+)::*mcr-1*	pET-28 a (+) encoding *mcr-1*	Kanamycin	This work
pET-28 a (+)::*mcr-1*$_{P198A}$	pET-28 a (+) encoding *mcr-1*$_{P198A}$	Kanamycin	This work
pET-28 a (+)::*mcr-1*$_{H478A}$	pET-28 a (+) encoding *mcr-1*$_{H478A}$	Kanamycin	This work
pSIM5-tet	Encodes the λ-Red recombinase genes (gam, bet and exo)	Tetracycline	B. Berghoff (Gift)
pKD4	Template plasmid for gene deletion	Amp/Kan	Addgene
pCP20	Encodes the yeast Flp recombinase gene	Amp/Cm	Addgene
pV163M	IncX4 plasmid	Kanamycin	Falgenhauer et al. 2016

Software
SPAdes
BLASTn
DNASTAR Lasergene 10.0 Core Suite
Magellan Data Analysis Software 7.2
Microsoft Office 2018
GraphPad Prism 5.01
Unicorn 5.20
Unipro UGENE v39.0.
Mega v10.8

Software
TMHMM-2.0
RoseTTAFold
Ramachandran Plot
pymol v1.7.4.5
ESPript3
XCalibur 3.1
LipidXplorer 1.2.8
ChemBioDraw Ultra 14.0
CorelDraw X7

Amp = Ampicillin

Kan = Kanamycin

Cm = Chloramphenicol

HRP = horseradish peroxidase

Restriction enzyme recognition sites are underlined
